# Impact and Cost-Effectiveness of Point-Of-Care CD4 Testing on the HIV Epidemic in South Africa

**DOI:** 10.1371/journal.pone.0158303

**Published:** 2016-07-08

**Authors:** Alastair Heffernan, Ella Barber, Ranjeeta Thomas, Christophe Fraser, Michael Pickles, Anne Cori

**Affiliations:** Department of Infectious Disease Epidemiology, Imperial College London, London, United Kingdom; British Columbia Centre for Excellence in HIV/AIDS, CANADA

## Abstract

Rapid diagnostic tools have been shown to improve linkage of patients to care. In the context of infectious diseases, assessing the impact and cost-effectiveness of such tools at the population level, accounting for both direct and indirect effects, is key to informing adoption of these tools. Point-of-care (POC) CD4 testing has been shown to be highly effective in increasing the proportion of HIV positive patients who initiate ART. We assess the impact and cost-effectiveness of introducing POC CD4 testing at the population level in South Africa in a range of care contexts, using a dynamic compartmental model of HIV transmission, calibrated to the South African HIV epidemic. We performed a meta-analysis to quantify the differences between POC and laboratory CD4 testing on the proportion linking to care following CD4 testing. Cumulative infections averted and incremental cost-effectiveness ratios (ICERs) were estimated over one and three years. We estimated that POC CD4 testing introduced in the current South African care context can prevent 1.7% (95% CI: 0.4% - 4.3%) of new HIV infections over 1 year. In that context, POC CD4 testing was cost-effective 99.8% of the time after 1 year with a median estimated ICER of US$4,468/DALY averted. In healthcare contexts with expanded HIV testing and improved retention in care, POC CD4 testing only became cost-effective after 3 years. The results were similar when, in addition, ART was offered irrespective of CD4 count, and CD4 testing was used for clinical assessment. Our findings suggest that even if ART is expanded to all HIV positive individuals and HIV testing efforts are increased in the near future, POC CD4 testing is a cost-effective tool, even within a short time horizon. Our study also illustrates the importance of evaluating the potential impact of such diagnostic technologies at the population level, so that indirect benefits and costs can be incorporated into estimations of cost-effectiveness.

## Introduction

Antiretroviral therapy (ART) substantially improves outcomes of human immunodeficiency virus (HIV) positive patients, particularly if initiated early [1]. But ART also dramatically reduces the risk of onwards transmission, and thus plays a role in prevention as well as treatment [2]. Following the accumulated evidence of this double benefit of ART, the World Health Organisation (WHO) has progressively updated its guidelines for initiation of ART in individuals diagnosed with HIV, gradually expanding the population for which ART initiation is recommended [3–6]. The remarkable expansion of ART availability across Sub-Saharan Africa, where 70% of worldwide infections occur [7], has led to a drop in AIDS-related deaths, which have decreased by 35% since peaking in 2005 [8]. In order to optimise the benefits of ART for the individual and the population, effective administration of the treatment cascade is essential. Improving the cascade implies early diagnosis, efficient linkage to care, timely ART initiation in those eligible for treatment, and regular follow-up to ensure adherence and sustained viral suppression. Yet attrition across the treatment cascade remains considerable, with only a quarter of HIV positive individuals in Sub-Saharan Africa estimated to be virally suppressed in 2012 [7].

There are numerous opportunities for losses throughout the treatment cascade, in particular when laboratory testing is involved. The latest WHO guidelines recommend immediate treatment, i.e. ART initiation, in all individuals living with HIV [6]. National guidelines often, however, still contain CD4 count thresholds above which treatment is not initiated (CD4 counts being used as a marker of immunodeficiency level). In South Africa, for example, treatment is available only for those with low CD4 cell counts (CD4≤500 cells/*μ*L), and is prioritised for those with even lower CD4 counts (CD4≤350 cells/*μ*L), severe disease and HIV/tuberculosis co-infection [9]. Until countries adopt the most recent guidelines, CD4 testing will continue to be carried out during treatment staging. Moreover, we anticipate that, even after immediate treatment protocols are formally adopted, CD4 cell counts will still be measured in resource limited settings in order to prioritise treatment for those most in need [6, 9].

In South Africa, CD4 testing facilities are generally centralised at hospital-based clinics within large urban areas [10]. A recent review of existing services showed that more than 50 clinics providing ART are 3-4 hours drive from the closest CD4 testing facility [10]. Consequently, turnaround time for a CD4 test can be a matter of weeks [10–13]. Moreover, the current system involves the patient repeatedly visiting the clinic, at least once for a blood draw and once to receive the CD4 test results. The time and cost (for the patient) of repeat travel to a distant clinic often leads to attrition [14, 15].

The development of point-of-care (POC) CD4 technology has been proposed as a way of decentralising laboratory services, thereby expanding access to, and reducing turnaround time for, CD4 testing. By delivering same day results, POC CD4 testing offers the potential for the CD4 cell count to be assessed at the time of HIV diagnosis. By reducing the number of clinic visits a patient is required to make, they are less likely to be lost to follow-up (LTFU) and eligible individuals may initiate treatment sooner. Several POC CD4 pilot studies have illustrated its promise for increasing retention within the treatment cascade [12, 13, 16–18].

While POC CD4 testing has the potential to overcome the poor retention currently experienced at this step within the treatment cascade, the associated costs may be high, and it is important to assess whether this approach is cost-effective. Hyle et al. used a Monte Carlo state-transition model to evaluate the costs and benefits of POC CD4 testing in Mozambique. Compared to laboratory CD4 testing, it was found to be a cost-effective way to improve individual patient outcomes in that setting [19]. This model, however, only captured the direct benefits (and associated costs) of improved life expectancy to the individual, and it did not incorporate the indirect benefits (and costs) of reduced onwards transmission within the population. Additionally, this paper considered only the impact of POC CD4 testing in a low income country with a less efficient programme of linkage to care, precisely a scenario in which a benefit would be expected from POC CD4 technology.

Dynamic transmission models allow capturing both direct and indirect effects of ART and provide a suitable means by which to evaluate the potential epidemiological impact of introducing POC CD4 testing. In this study, we used mathematical modelling to evaluate the cost-effectiveness of introducing POC CD4 testing in South Africa, compared to laboratory CD4 testing as is currently used. We considered three HIV care contexts corresponding to different levels of HIV care delivery that may be delivered in South Africa in the near future. For each of these, we evaluated whether the introduction of POC CD4 testing was predicted to be cost-effective, over different time horizons. We used a probabilistic approach to assess whether our findings were robust to uncertainties in future costs as well as in epidemiological parameters.

## Methods

### Epidemiological model structure

We extended a previously developed dynamic, deterministic model of heterosexual HIV transmission in South Africa [20]. In brief, that model simulated a heterosexual population representing South African adults aged 15-49 years from the start of the HIV epidemic. The population was divided by gender and sexual risk, with males additionally divided by circumcision status. Births and non-HIV related mortality were included in the model. Sexual mixing was assortative by level of risk. HIV infected individuals were classified in four CD4 count categories (in units of cells/*μ*L: CD4 ≥ 500, 350 ≤ CD4 < 500, 200 ≤ CD4 < 350, CD4 < 200). HIV infectivity depended on HIV disease stage and whether the individual was on ART or not; men who were circumcised were assumed to have lower susceptibility to infection. Full details are in Cori et al. [20].

Our extended model incorporated a more detailed HIV treatment cascade process, and was calibrated to the most recently available HIV prevalence estimates for South Africa [21]. Specific additions were the division of the process between HIV testing and entering ART care to include a more realistic CD4 staging step, as well as the inclusion of first line ART treatment failure with the possibility of second line ART (see S1 Supporting Information for detail).

Once an individual tested HIV positive, they could be CD4 staged (which combines CD4 testing and receiving results). If not eligible according to national guidelines at that time, they entered pre-ART care, otherwise they initiated ART. ART failure could occur, leading to initiation on the more expensive second line ART. Individuals could be LTFU at any stage. Those LTFU during CD4 staging re-entered the diagnostic cascade to undertake treatment at a rate lower than that of initial entry, chosen to ensure numbers on ART were greater than 2 million by 2012 [22]. Those LTFU while on ART could subsequently reinitiate treatment.

### Model parameterisation

#### Basic model parameters and calibration

The epidemiological parameters were taken from the literature where possible (see S1 Supporting Information). Model parameters anticipated to influence the epidemiology were varied, see Table A in S1 Supporting Information [20]. Two million parameter sets were generated using Latin hypercube sampling with uniform prior ranges [23]. Parameter sets which generated prevalence values within twice the UNAIDS confidence intervals from 1993 to 2013 [21], and in which at least two million individuals were on ART by 2012 [24, 25], were considered calibrated and used in the cost-effectiveness analysis.

#### CD4 staging parameters

To parameterise CD4 staging for both POC and laboratory CD4 testing, we carried out a meta-analysis of clinical and observational trial data [12–14, 16–18, 26, 27]. Not all studies reported matching data between POC and laboratory CD4 testing arms. As such, odds ratios could not be always be computed. Instead, the proportions that successfully received CD4 results following a CD4 test ($p_{CD4results}^{POC/lab}$) and successfully initiated ART following receipt of CD4 results ($p_{ART-initiate}^{POC/lab}$) were individually calculated by pooling data from the trials using a DerSimonian-Laird random effects model [28] (for further details see Table C and S1.3.4 in S1 Supporting Information). The proportions were varied independently within the 95% confidence intervals derived in the meta-analysis.

#### Costs and DALYs

Following Eaton et al. [29], each compartment in the model had an associated healthcare cost. In addition, HIV testing, CD4 testing, and end-stage care also incurred costs. All costs were in US$ 2015. A programme markup of 1.5× was added to all non-antiretroviral (ARV) expenditure to account for fixed costs. ARV costs were marked up by 1.2× to account for logistics. ART costs and non-ART costs were varied (independently) between -20% and 20% of their value (including markup) to allow for future price changes. Economic parameters are described in S1 Supporting Information; for a list of costs used (adjusted for inflation) see Table E in S1 Supporting Information. Disability adjusted life years (DALYs) were calculated using standard disability weightings for HIV, see Table D in S1 Supporting Information. All cost differences and DALYs averted in future years were discounted at between 0.1% and 7.0% (varied in sensitivity analysis).

#### Care contexts

We assessed the potential impact and cost-effectiveness of POC CD4 testing compared to laboratory testing within three distinct care contexts. These were designed to reflect potential future HIV care contexts in the South African healthcare system. The contexts considered were:

- Current care (CC) context: after 2015 annual HIV testing numbers continued to increase in line with population growth (from an estimated number of HIV tests of 10 million in mid-2011 [22]); return to ART is kept at the same level as before 2015 (a rate, varied in sensitivity analysis, such that between 5% and 80% of those with CD4 < 200 cells/*μL* return to ART within one year of drop-out); no further changes in national guidelines for ART initiation.
- Enhanced counselling and testing (ECT): from 2015 the whole population tested annually for HIV; annual rates of return from LTFU were approximately 95%; no further changes in national guidelines for ART initiation.
- Universal test and treat (UTT): as ECT context but expansion of ART to all HIV positive individuals; patients still received a CD4 test prior to ART initiation for clinical assessment and, therefore, POC CD4 testing also improves linkage to care relative to laboratory CD4 testing in this context.

The first context represented a continuation of current HIV care efforts. The second assumed extreme improvements in both HIV testing efforts and retention in care. The third further assumed that South Africa adopted immediate treatment following the most recent WHO guidelines [6]. The second and third contexts may seem unrealistically optimistic but together with the first allowed us to explore the impact of POC CD4 in a wide range of care contexts.

### Plan of analysis

For each of the above care contexts, two simulations were run: one with the implementation of POC CD4 testing (from 2015 onwards) and a comparator with laboratory testing. For each care context, the additional cost of introducing POC CD4 testing was estimated, along with the infections averted and DALYs averted for one and three year time horizons. A probabilistic approach to cost-effectiveness analysis was carried out by varying the CD4 staging and economic parameters (costs and discount rate) for each of the epidemiological parameter sets generated in calibration. Specifically, 250 CD4 and cost parameter sets were obtained by uniform sampling within ranges shown in Table E in S1 Supporting Information and the results of the meta-analysis, see below. Incremental cost-effectiveness ratios (ICER = cost incurred/DALYs averted) were derived for one and three year projections for all combined parameter sets and compared to South Africa’s GDP per capita ($6,478 [30]) to assess cost-effectiveness [31]. Ranges for the proportion of infections averted were calculated in the same way.

A sampling-based approach was used to assess sensitivity of the infections averted and ICERs to input parameter variation [32]. Using the complete model outputs, multivariate linear regression models were constructed for both infections averted and ICERs as functions of the varied parameters. Appropriateness of using linear models was checked by ensuring model *R*^2^ were large. Standardised regression coefficients are reported in S2 Supporting Information, along with the proportion of variance explained by each parameter. Since parameters are sampled from a Latin hypercube, they are uncorrelated and thus the standardised regression coefficients measure one-way sensitivity (they are equal to Pearson’s correlation coefficients) [32]. The parameters which explain more than 10% of the variance are reported here, and were used to construct linear models for ICERs and infections averted in each context, see S2 Supporting Information.

## Results

### Calibration and meta-analysis

**Fig 1:**
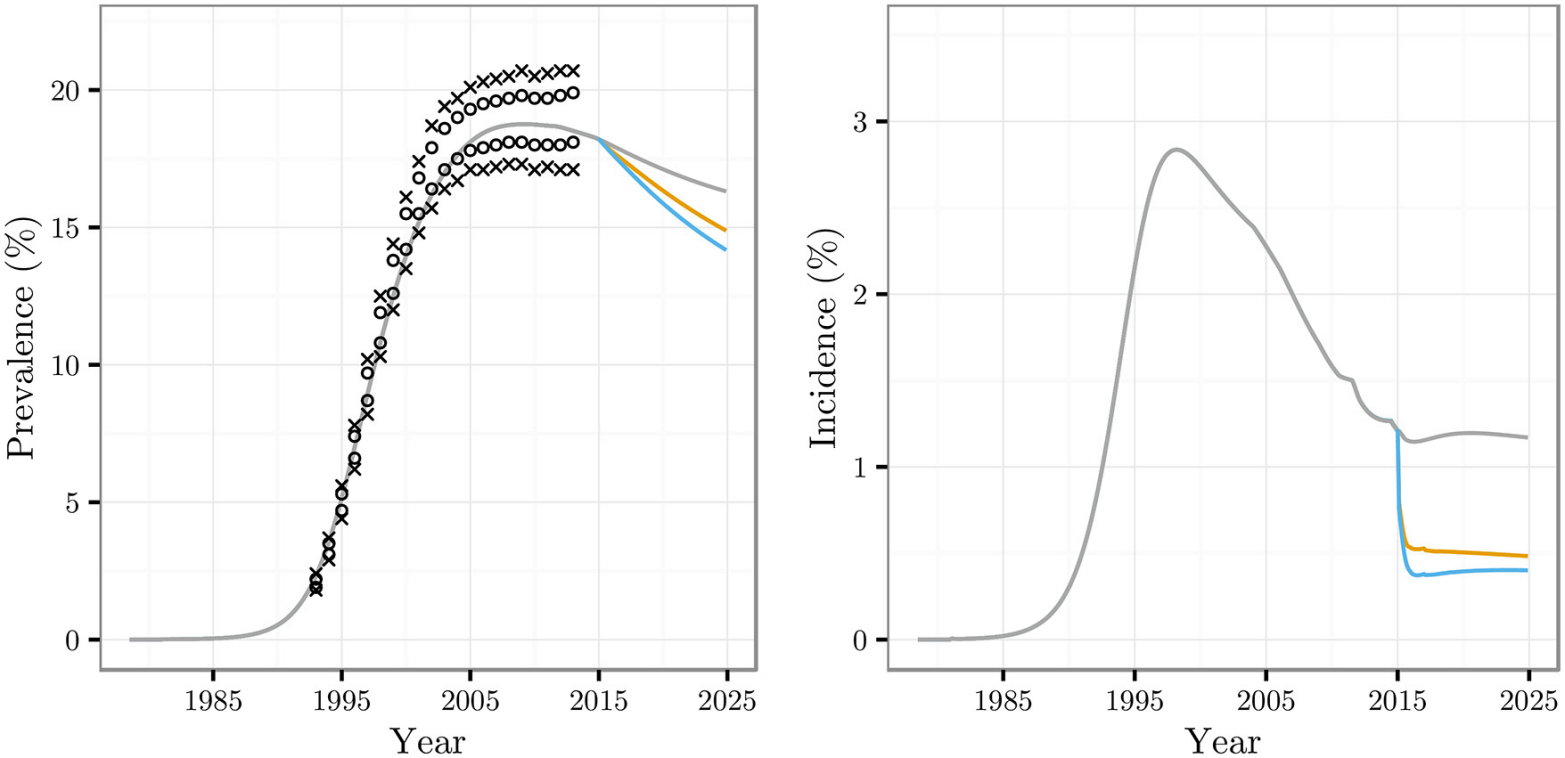
Prevalence and incidence curves. Prevalence and incidence for all three care contexts, for an arbitrarily chosen calibrated epidemiological parameter set. For all parameter sets see Fig C in S1 Supporting Information. The colours correspond to (from top to bottom on both panels): grey—current care (CC) context, orange—enhanced counselling and testing (ECT), blue—universal test and treat (UTT). Also shown on the left panel are the confidence intervals of the UNAIDS prevalence estimates (inner circles) and twice the confidence intervals (outer crosses).

Results of the meta-analysis quantifying the differences in CD4 staging between POC and laboratory CD4 testing are shown in Table 1. Prevalence and incidence over time for the three care contexts are shown in Fig 1, for one of the 71 parameter sets that satisfied calibration requirements. Prevalence decreases in all three care contexts, though more rapidly in the enhanced programmes after 2015; incidence drops rapidly after introduction of enhanced measures in ECT and UTT contexts, after which it plateaus.

**Table 1:** Results of meta-analysis of CD4 staging for POC CD4 and laboratory testing (95% confidence intervals shown in brackets).

Parameter	Lab CD4 testing	POC CD4 testing
Proportion receiving CD4 results of those CD4 tested (%)	57.7 (41.8 - 72.9)	90.8 (82.2 - 96.8)
Proportion initiating ART of those receiving CD4 results (and ART eligible) (%)	51.5 (45.1 - 57.9)	62.2 (47.5 - 75.9)

### ICERs, infections averted and costs

**Fig 2:**
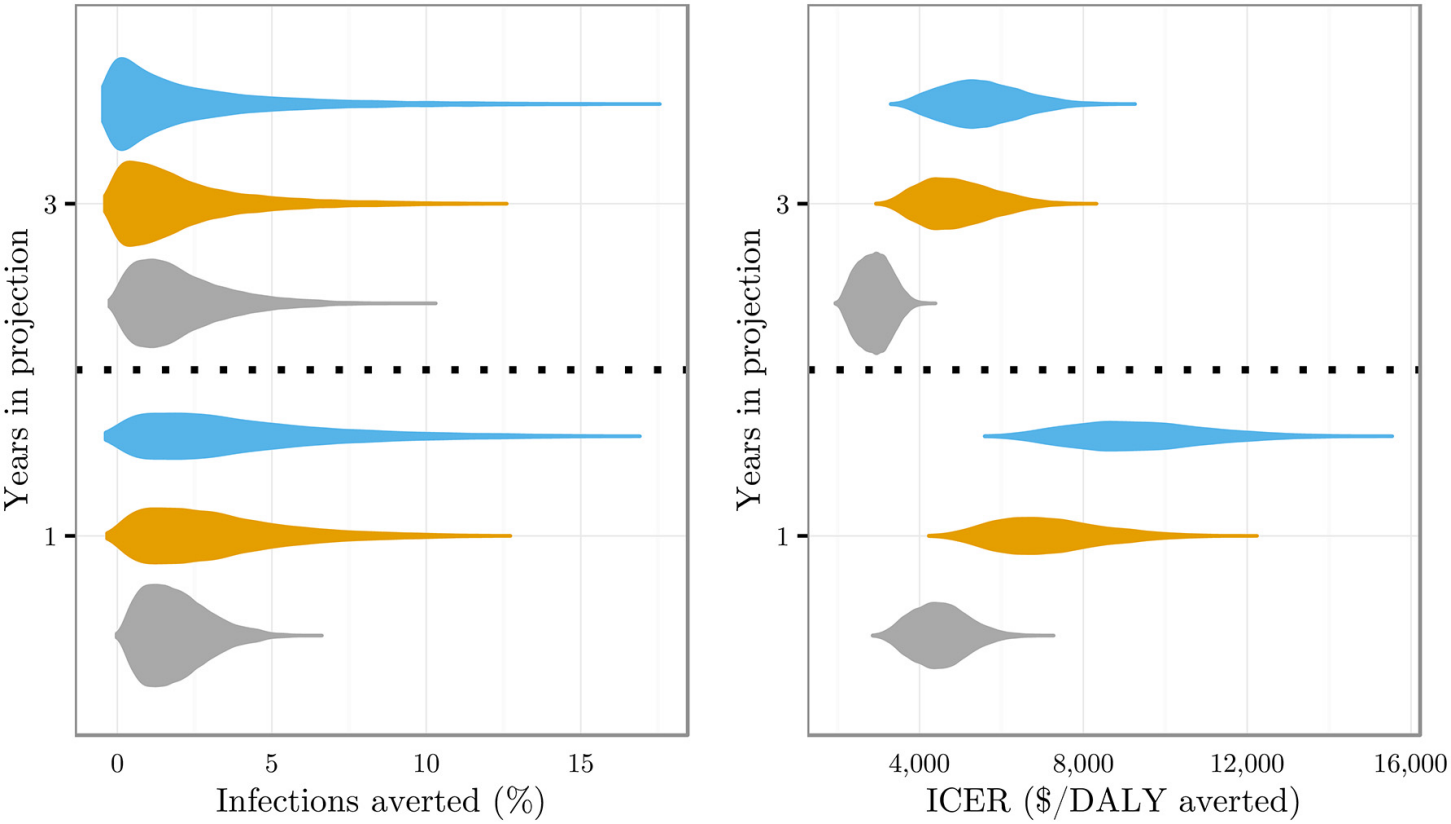
Violin plots showing infections averted and ICERs when introducing POC CD4 testing compared to laboratory testing in each care context. Left plot shows infections averted across one and three year projections. The right plot shows ICERs. The colours correspond to (from bottom to top in each year): grey—current care (CC) context, orange—enhanced counselling and testing (ECT), blue—universal test and treat (UTT). Note: when linkage to care is high but the effectiveness of ART in reducing infectivity is low (close to 50%), patients live longer and have more opportunity for transmission than would be the case without the introduction of POC CD4 testing. This results in the observed negative infections averted but, since more individuals are on treatment, DALYs averted (and so ICERs) are still positive.

The projections of infections averted and ICERs are shown in Fig 2 for one and three year time horizons. The projected proportions of infections averted has a wide distribution whose width increases over time; the median slightly decreases from one to three year projections in all contexts. Cost-effectiveness is higher over three years than over one year; the most cost-effective programme is the CC context, followed by the ECT then UTT contexts.

In all three contexts, introducing POC CD4 testing led to infections averted over a one year horizon compared to laboratory CD4 testing: 1.72% (95% confidence interval: 0.36—4.31%) infections averted in CC context, 2.65% (0.17—8.78%) in ECT context, 3.09% (0.11—11.47%) in UTT context. Over the same period, DALYs averted (which reflect both infections averted and the improved health of those additionally on treatment) were 0.02m DALYs (0.01 million—0.04 million DALYs) in CC context, and 0.03m DALYs (0.02 million—0.05 million DALYs) in both ECT and UTT contexts.

After 1 year the total additional cost from introducing POC CD4 testing was $0.11 billion in CC context, $0.22 billion in ECT context and $0.29 billion in UTT context (see Table I in S2 Supporting Information). Note each of these contexts differed greatly in total cost (with UTT being the most expensive context) and the costs presented above represent only the additional costs from introducing POC CD4 in each context. Results for the three year projections are presented in S2 Supporting Information.

### Cost-effectiveness analysis

**Fig 3:**
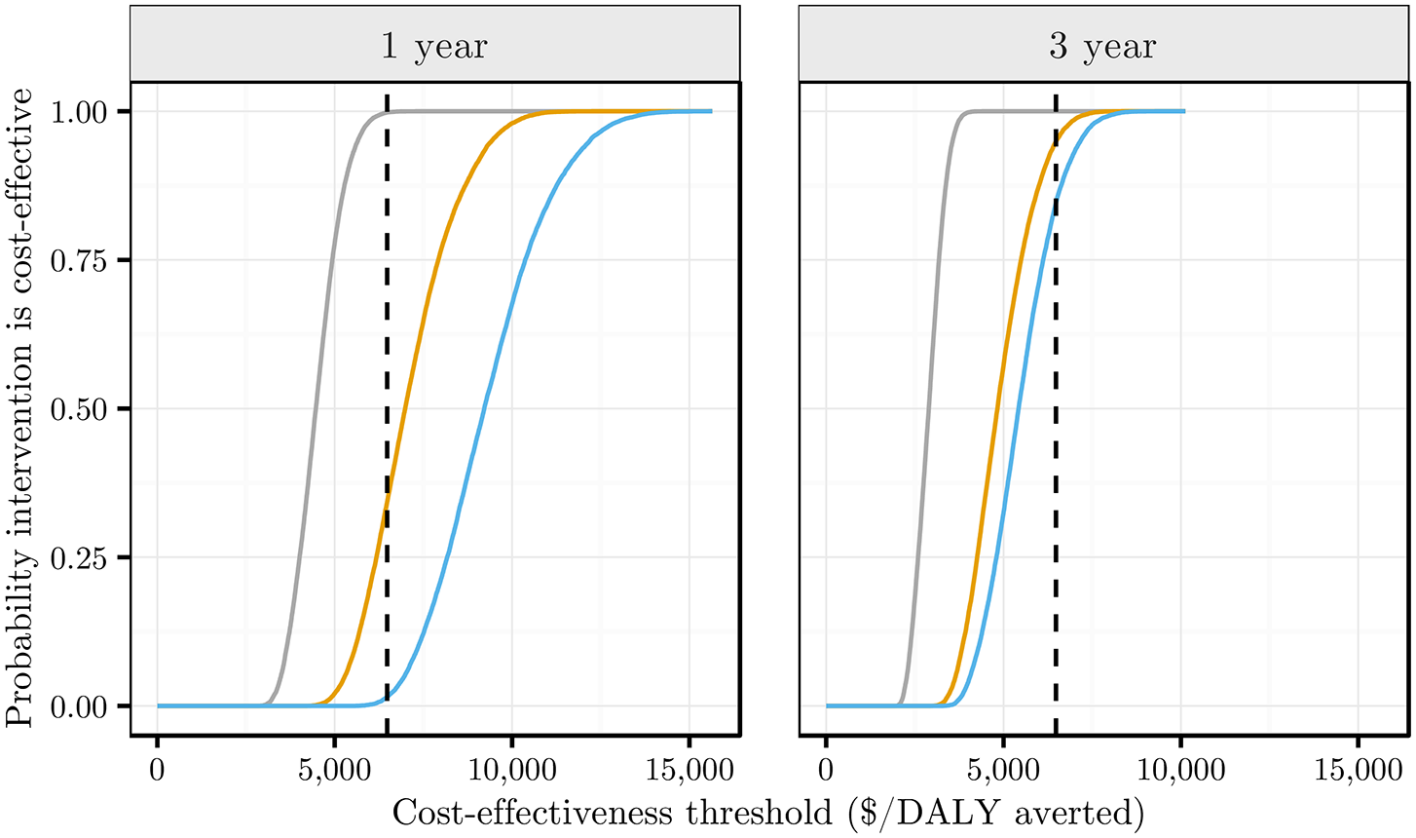
Cost-effectiveness acceptability curves. These curves show the probability that introduction of POC CD4 testing compared to laboratory CD4 testing is cost-effective at a range of decision rule thresholds for the 1 year projection (left) and 3 year projection (right). The colours correspond to (left to right within each plot): grey—current care (CC) context, orange—enhanced counselling and testing (ECT), blue—universal test and treat (UTT). The dashed line shows South African GDP per capita.

Cost-effectiveness acceptability curves (CEAC) are shown in Fig 3 (for cost-effectiveness plane graphs see Fig D in S2 Supporting Information). The CEAC curves show that introducing POC CD4 testing into the CC context is likely to be cost-effective after one year at the willingness-to-pay threshold of South African GDP; the ECT and UTT contexts are only likely to be cost-effective after three years.

The probability of each of the three interventions being cost-effective after 1 year when compared to the threshold of South Africa’s GDP per capita ($6,478 [30]) were: in the CC context—99.8%, in the ECT context—34.3% and in the UTT context—1.5%. After 3 years the probabilities of being cost-effective were: CC—100%, ECT—94.9% and UTT—84.5%. When using a threshold of 3 times GDP per capita, introducing POC CD4 testing was cost-effective in all three care contexts at one and three year time scales. Additional details of the results are given in S2 Supporting Information.

### Sensitivity analysis

Full linear regression results for ICERs and infections averted are shown in S2 Supporting Information, specifically Table F and Table G. Adjusted *R*^2^ values are high, demonstrating appropriateness of the linear models. The proportion of the variance explained by each parameter can, therefore, be used to quantify the extent to which each parameter drove the variation in ICERs and infections averted.

In short, the projected epidemiological impact due to the introduction of POC CD4 testing varied due to assumptions around treatment effectiveness, and the improvement in the proportion receiving their CD4 test results (and hence remaining in care) once POC CD4 testing was introduced. In the CC context, the improvement in linkage due to the introduction of POC CD4 testing was the key determinant of infections averted. Conversely, in the ECT and UTT contexts, the key determinant was the reduction in infectivity when on ART, due to the large number of individuals on ART.

Variation in ICERs was determined by several factors. In all contexts it was sensitive to the increase in the proportion receiving their CD4 test results through the introduction of POC CD4 testing. In terms of costs, the relative importance of ART cost and POC CD4 test cost varied by time horizon and care context. In the CC context, over one and three years, the ICER was more sensitive to costs associated with ART than with POC CD4 test costs. In ECT and UTT contexts, the one year ICERs were largely determined by POC CD4 test costs, because of the high volume of CD4 tests in the first year. After three years ART costs became the main driver of ICER variation.

## Discussion

In this study we have examined the epidemiological impact and cost-effectiveness of introducing point-of-care (POC) CD4 testing, in place of existing laboratory CD4 testing, in South Africa, for three different HIV care contexts that cover the range of possible ways that HIV care may evolve in the near future. Across all contexts considered, POC CD4 testing results in 1.7-3.1% of new HIV infections averted during the first year. After one year in the current care (CC) context, which most closely represents the current care situation in South Africa, the introduction of POC CD4 testing has a 99.8% probability of being cost-effective compared to the threshold of South African GDP per capita ($6,478), with a median ICER of $4,468/DALY averted. Cost-effectiveness increases over time, so that the probability it is cost-effective is 100% after three years. In the two care contexts with more intensive HIV testing and linkage to care (enhanced counselling and testing (ECT) and universal test and treat (UTT)), the median ICER over the first year is $6,986/DALY averted and $9,215/DALY averted respectively, and introducing POC CD4 testing is unlikely to be cost-effective over one year at the threshold of South African GDP per capita. In both contexts, however, the probability that the intervention is cost-effective (given the willingness-to-pay threshold of South African per-capita GDP) is higher in the three year than in the one year scenario: within three years the probability of being cost-effective is 84.5% in the UTT context, and higher in the intermediate (ECT) context.

Given the past delays in adopting and implementing changes in WHO guidelines, and the financial and infrastructural challenges associated with moving to ART irrespective of CD4 count in a country with such high prevalence, the CC context is likely to remain the most representative context in the short term. In that context POC CD4 testing was found to be cost-effective even over a one year horizon. Anticipated expansions in treatment and testing may make the ECT and UTT contexts more representative of future HIV care in South Africa. Our results show that the introduction of POC CD4 testing is very likely to be cost-effective over three years in these contexts.

Hyle et al. [19] previously found that introducing POC CD4 testing was cost-effective in the context of the HIV epidemic in Mozambique, with the threshold for ART initiation at CD4< 250 cells/*μ*L, with an ICER of $500 per year of life saved. They found that POC CD4 testing was no longer cost-effective in that setting when linkage to care was improved. This is consistent with our finding that cost-effectiveness is notably lower in the ECT and UTT contexts, in which numbers HIV testing was significantly increased compared to the CC context. This concordance in findings is notable given differences between the studies: our analysis considers shorter time horizons than Hyle et al., incorporates indirect effects and is set within a higher income setting.

The results demonstrate that POC CD4 testing is more likely to be cost-effective when introduced into less comprehensive HIV care contexts: firstly, the probability of being cost-effective is higher in the current care context compared to the enhanced (ECT and UTT) contexts, in which additional HIV testing occurs and retention in care is improved. Secondly, cost-effectiveness increases (the ICER decreases) when the improvement in proportion receiving their results (in the POC versus laboratory CD4 testing strategy) is increased. This may occur in situations where the proportion successfully CD4 staging with laboratory testing is particularly low.

This supports a more targeted approach to its introduction. Glencross et al. recently reviewed the provision of CD4 services at a subnational level across South Africa [10], identifying gaps in service provision in remote areas. They proposed an integrated tiered service delivery model that allows an extension of services to rural areas while making use of existing clinic infrastructure. A combination of POC CD4 testing and decentralised laboratory tiers was found to be a cheaper way to expand diagnostic capacity as opposed to offering widespread POC CD4 testing [10]. Prioritising implementation to specific settings could further reduce costs through hierarchical support across tiers, for example through increased efficiency of training and quality control.

One strength of this study is the use of a dynamic transmission model, which captures the prevention of future HIV infections through reduced prevalence and the associated future healthcare savings. This approach could be used in future work to estimate the population-level impact of POC CD4 testing in other settings, especially those with less efficient linkage to care. Another strength is that a previous iteration of the model was part of a model comparison exercise in which the projected reduction in HIV incidence in South Africa due to an ART intervention was, at least in the short-term, consistent with other mathematical models [33]. In addition, our model is fitted to, and reproduces, the most recent UNAIDS HIV prevalence trends for South Africa. A difficulty in parameterising the model was the lack of data concerning re-entry into care after ART drop-out. To circumvent this issue, we fully explored the uncertainty in our analysis and found that it did not influence the outcomes within our timeframe. Moreover, our use of short timeframes ensures we are confident that our findings are robust, since the use of short term incidence measures in modelling studies has been shown to be very robust to changes in model structure [33]. Finally, quantifying the impact of CD4 testing on linkage to care was a key part of the parameterisation and was achieved by a meta-analysis.

## Conclusions

This study shows that in the middle-income setting of South Africa, introduction of POC CD4 testing is likely to be cost-effective (under the threshold of GDP per capita), in comparison to existing laboratory CD4 testing, and that cost-effectiveness increases over time. CD4 counts may stop being used to assess eligibility for ART initiation in South Africa within the next couple of years; however, we found that the introduction of POC CD4 testing in the current care context was very likely to be cost-effective within one year. Moreover, CD4 testing is likely to remain a widespread tool for clinical staging of HIV positive patients before ART initiation. Given this, our findings suggest that even if ART is expanded to all HIV positive individuals in the near future, and HIV testing efforts are substantially increased, POC CD4 testing is still a cost-effective tool. Our study also illustrates the wider importance of evaluating the potential impact and cost-effectiveness of such diagnostic technologies at the population level in the future.

## Supporting Information

S1 Supporting Information Additional model information. S1 Supporting Information contains an overview of the HIV transmission model, a list of the model equations and parameters, a discussion of the model structure and parameterisation, and details of the economic analysis.
(PDF)

S2 Supporting Information Additional results. S2 Supporting Information contains additional results tables and graphs, including results of the linear analysis used to assess the key predictors of cost-effectiveness of introducing POC CD4 testing.
(PDF)
