# Supplementary material for: Impact and Cost-Effectiveness of Point-Of-Care CD4 Testing on the HIV Epidemic in South Africa
Source: PLoS One. 2016 Jul 8;11(7):e0158303. doi: 10.1371/journal.pone.0158303 (PMC4938542; doi:10.1371/journal.pone.0158303)
Supplement: S1 Supporting Information — contains an overview of the HIV transmission model, a list of the model equations and parameters, a discussion of the model structure and parameterisation, and details of the economic analysis. (PDF) [file pone.0158303.s001.pdf]

S1 Supporting Information. Additional model information

This section provides an overview of the model, with a diagram of the model structure, the differential equations that define it and tables of quantities in the model. The model parameters are then discussed in greater depth with an explanation of the model's structure.

S1.1 Overview of the model

The model is a modified version of that described in [1]. The model structure for women is shown in Fig A (the structure for men is similar with the addition of compartments for circumcision, see Fig B). The model was coded in C++, and differential equations were solved using a Runge-Kutta 4 solver, with a time-step $dt = 0.002$ years.

The model consists of a set of differential equations, equations (1)-(18) (see S1.2 below), equivalent to Fig A and Fig B. There are a number of state variables (S, P, I, H, C, L, J, T, A, LA, A_{SL}, LA_{SL}). S represents susceptible individuals, P represents acute HIV infection, and the remaining state variables, corresponding to different points in and out of the HIV care cascade, are defined by the text on the right hand side of Fig A (the text corresponds to the state variable in the given row of the figure). Each state variable has subscripts giving the gender ($a = m, f$) and sexual risk group ($i = 1, 2, 3$, corresponding to higher, middle and lower risk groups respectively). For men who are HIV negative (S_m) or in acute infection (P_m) there is an additional middle subscript that denotes circumcision status (u: uncircumcised, p: planning circumcision, h: healing post-circumcision, c: circumcised). Amongst HIV-positive individuals post-acute infection, there is an additional superscript $k = 1, 2, 3, 4$ denoting the CD4 stage of the individual (CD4 $\geq$ 500, 350$\leq$ CD4 $<$ 500, 200 $\leq$ CD4 $<$ 350, CD4 $<$200 cells/μL respectively). The subscript SL refers to second-line ART.

The model can be summarised as follows. It represents individuals aged 15 and over. The number of new individuals, aged 15, entering the sexually active population at a given time is taken to depend on the population size and the adult birth rate 15 years beforehand. For men, a fixed proportion are circumcised prior to entry in the model. For each gender, a fraction of individuals enters in each risk group i. Death rates are adjusted to reflect projections of mortality for the country as a whole.

Susceptible, uncircumcised men S_{mui} can become circumcised once sexually active. During the circumcision healing phase, individuals can become infected with HIV, but with a modified risk of HIV acquisition that accounts for the trade-off between increased biological susceptibility and reduced sexual activity during this time [1]. Once healed, circumcised men have reduced susceptibility to infection. However, once individuals become infected with HIV the model no longer tracks their circumcision status, as circumcision is assumed not to affect infectivity.

The epidemic is seeded with 0.1% of the male population taken to be HIV-positive (added to compartment P). HIV infected men infect women according to the force of infection FOI_{fi}, and vice-versa. If an individual becomes HIV-infected, there is a short period of acute infection with increased infectivity. During this time, due to the window period of HIV tests, if they undergo an HIV test they will receive a negative result, and, therefore, in the model they will not link to care. At the end of acute infection individuals enter one of four CD4 count categories ($\geq$500, 350-500, 200-350, $<$200 cells/μL).

They then progress sequentially from that initial CD4 category to lower categories. Individuals may die from AIDS-related illness only once they reach CD4 $<$ 200 cells/μL. In South Africa the national ART programme was launched in 2004 [2]. In the model we thus assume HIV testing starts in 2004, after which those testing positive for HIV can enter care and initiate ART, if eligible according to the then-current South African guidelines [3–6]. ART reduces infectivity. ART also either reduces the rate of disease progression or halts it entirely, if treatment is initiated early enough. Individuals on ART may be LTFU and later return to ART. They may also experience treatment failure and progress to second line ART.

S1.2 Model equations and parameters

Equations (1)-(18) define the model. Parameters that vary with time are shown with time dependence. Equations (1) to (8), defining the dynamics of the susceptible and acute infection compartments (S and P), are unchanged from [1]. Equations (9) to (18) show the remaining model equations, describing how HIV-positive individuals test, enter care, initiate ART and potentially are lost to follow-up (LTFU).

Fig A. Structure of model for women. Red arrows indicate losses to follow-up. Rates corresponding to green arrows are equal either to the indicated rate or to zero, depending on the CD4 threshold for ART initiation in place at a particular time. See text for detail on notations. Non AIDS-related deaths are not shown in this diagram for simplicity.

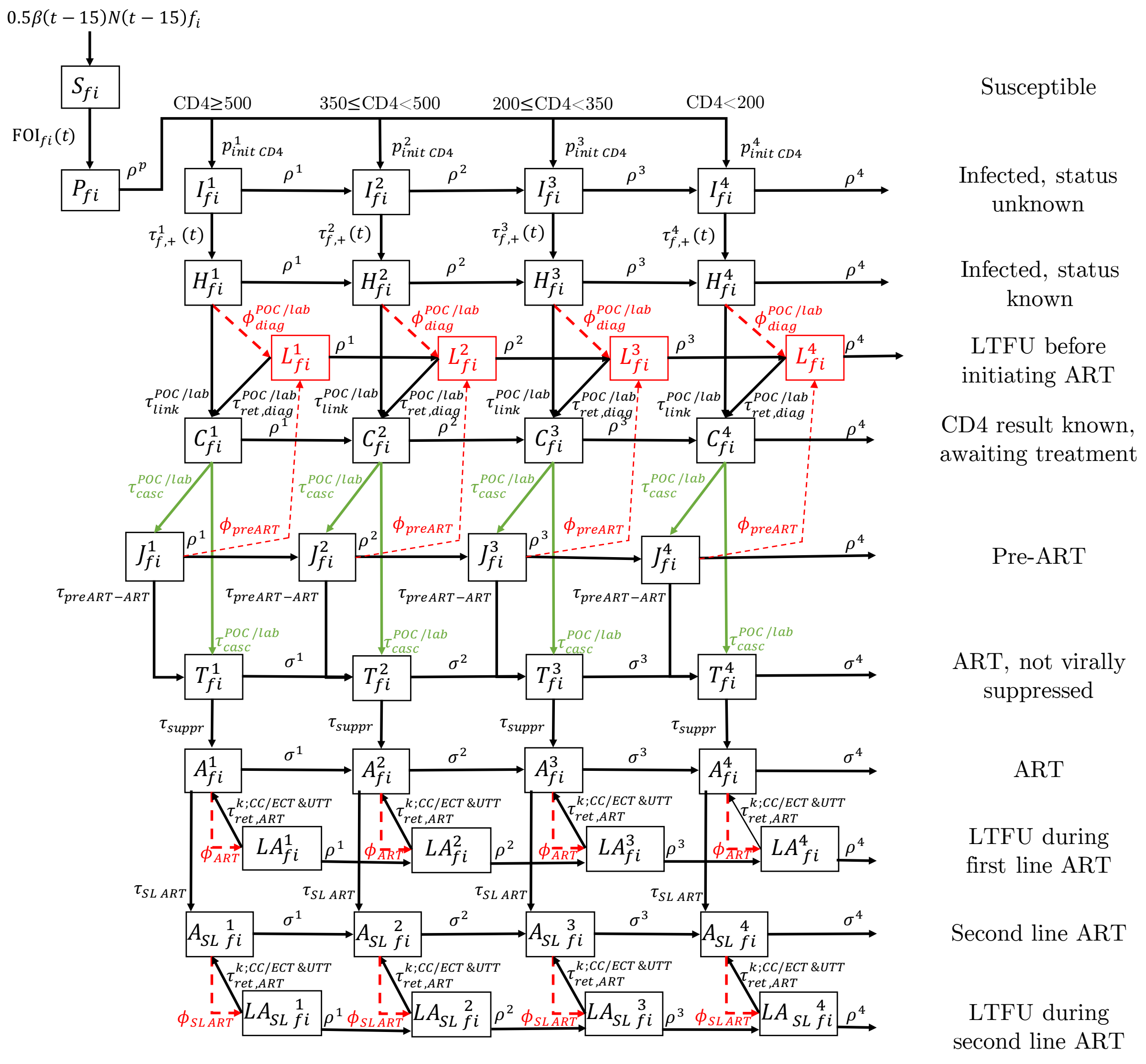

Fig B. Variation of model structure for men. Shown are the susceptible and acute infection compartments for men. After progressing from acute infection to chronic infection (compartments I^k_{ai}) the model is identical to Fig A.

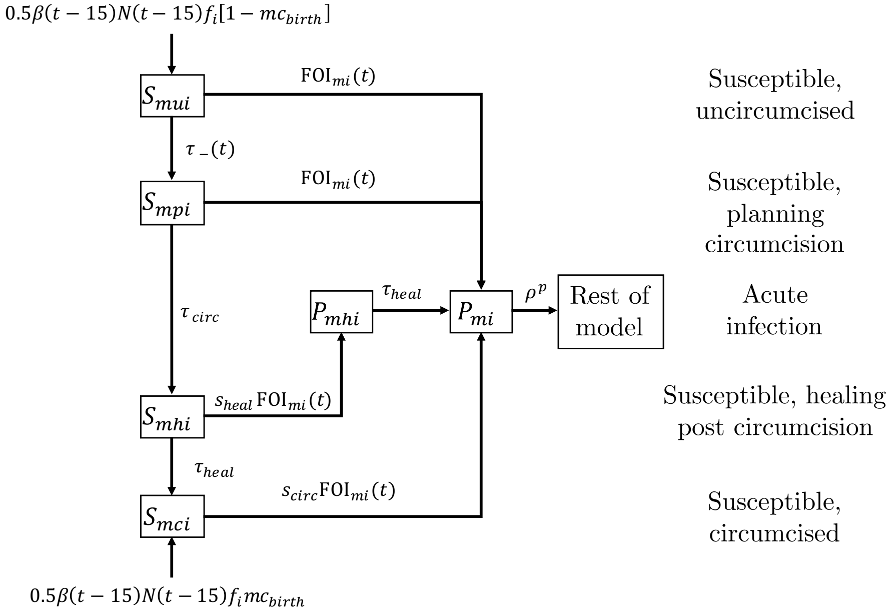

For ease of reading, state parameters (which all vary with time) are not shown with explicit time dependence. In general ϕ refers to a LTFU rate. A POC or lab superscript means the parameter is different for POC CD4 and laboratory CD4 test type respectively. Finally parameters with superscript CC or ECT/UTT vary according to the context being modelled:

$$\frac{dS_{mui}}{dt} = 0.5\beta(t-15)N(t-15)f_i\left[1-mc_{birth}\right] - \left[\mathrm{FOI}_{mi}(t) + \tau_-(t) + \mu(t)\right]S_{mui}, \quad (1)$$

$$\frac{dS_{mpi}}{dt} = \tau_-(t)S_{mui} - \left[\mathrm{FOI}_{mi}(t) + \tau_{circ} + \mu(t)\right]S_{mpi}, \quad (2)$$

$$\frac{dS_{mhi}}{dt} = \tau_{circ}S_{mpi} - \left[s_{heal}\mathrm{FOI}_{mi}(t) + \tau_{heal} + \mu(t)\right]S_{mhi}, \quad (3)$$

$$\frac{dS_{mci}}{dt} = 0.5\beta(t-15)N(t-15)f_i mc_{birth} + \tau_{heal}S_{mhi} - \left[s_{circ}\mathrm{FOI}_{mi} + \mu(t)\right]S_{mci}, \quad (4)$$

$$\frac{dS_{fi}}{dt} = 0.5\beta(t-15)N(t-15)f_i - \left[\mathrm{FOI}_{fi}(t) + \mu(t)\right]S_{fi}, \quad (5)$$

$$\frac{dP_{mhi}}{dt} = \mathrm{FOI}_{mi}(t)s_{heal}S_{mhi} - \left[\tau_{heal} + \mu(t)\right]P_{mhi}, \quad (6)$$

$$\frac{dP_{mi}}{dt} = \mathrm{FOI}_{mi}(t)\left[S_{mui} + S_{mpi} + s_{circ}S_{mci}\right] + \tau_{heal}P_{mhi} - \left[\rho^p + \mu(t)\right]P_{mi}, \quad (7)$$

$$\frac{dP_{fi}}{dt} = \mathrm{FOI}_{fi}(t)S_{fi} - \left[\rho^p + \mu(t)\right]P_{fi}, \quad (8)$$

$$\frac{dI^k_{at}}{dt} = \rho^p p^k_{initCD4} + \rho^{k-1}I^{k-1}_{ai} - \left[\tau^{k;CC/ECT\&UTT}_{a,+}(t) + \rho^k + \mu(t)\right]I^k_{ai}, \quad (9)$$

$$\frac{dH^k_{ai}}{dt} = \tau^{k;CC/ECT\&UTT}_{a,+}(t)I^k_{ai} + \rho^{k-1}H^{k-1}_{ai} - \left[\tau^{POC/lab}_{link} + \phi^{POC/lab}_{diag} + \rho^k + \mu(t)\right]H^k_{ai}, \quad (10)$$

$$\frac{dC^k_{ai}}{dt} = \tau^{POC/lab}_{link}H^k_{ai} + \tau^{k,POC/lab;CC/ECT\&UTT}_{ret,diag}L^k_{ai} + \rho^{k-1}C^{k-1}_{ai} - \left[\tau^{POC/lab}_{casc} + \rho^k + \mu(t)\right]C^k_{ai}, \quad (11)$$

$$\frac{dJ_{ai}^k}{dt} = \left[1 - \delta_{CD4-thresh}^k(t)\right] \tau_{casc}^{POC/lab} C_{ai}^k + \rho^{k-1} J_{ai}^{k-1} - \left[\delta_{CD4-thresh}^k(t) \tau_{preART-ART} + \phi_{preART} + \rho^k + \mu(t)\right] J_{ai}^k, \tag{12}$$

$$\frac{dL_{ai}^k}{dt} = \phi_{diag}^{POC/lab} H_{ai}^k + \phi_{preART} J_{ai}^k + \rho^{k-1} L_{ai}^{k-1} - \left[\tau_{ret,diag}^{k,POC/lab;CC/ECT\&UTT} + \rho^k + \mu(t)\right] L_{ai}^k, \tag{13}$$

$$\frac{dT_{ai}^k}{dt} = \delta_{CD4-thresh}^k(t) \tau_{casc}^{POC/lab} C_{ai}^k + \delta_{CD4-thresh}^k(t) \tau_{preART-ART} J_{ai}^k + \sigma^{k-1} T_{ai}^{k-1} - \left[\tau_{suppr} + \sigma^k + \phi_{ART} + \mu(t)\right] T_{ai}^k, \tag{14}$$

$$\frac{dA_{ai}^k}{dt} = \tau_{suppr} T_{ai}^k + \tau_{ret,ART}^{k;CC/ECT\&UTT} LA_{ai}^k + \sigma^{k-1} A_{ai}^{k-1} - \left[\phi_{ART} + \tau_{SL\,ART} + \sigma^k + \mu(t)\right] A_{ai}^k, \tag{15}$$

$$\frac{dLA_{ai}^k}{dt} = \phi_{ART} A_{ai}^k + \rho^{k-1} LA_{ai}^{k-1} - \left[\tau_{ret,ART}^{k;CC/ECT\&UTT} + \rho^k + \mu(t)\right] LA_{ai}^k, \tag{16}$$

$$\frac{d\,{A_{SL}}_{ai}^k}{dt} = \tau_{SL\,ART} A_{ai}^k + \tau_{ret,ART}^{k;CC/ECT\&UTT}\, {LA_{SL}}_{ai}^k + \sigma^{k-1}\, {A_{SL}}_{ai}^{k-1} - \left[\phi_{SL\,ART} + \sigma^k + \mu(t)\right] {A_{SL}}_{ai}^k\,, \tag{17}$$

$$\frac{d\,{LA_{SL}}_{ai}^k}{dt} = \phi_{SL\,ART}\, {A_{SL}}_{ai}^k + \rho^{k-1}\, {LA_{SL}}_{ai}^{k-1} - \left[\tau_{ret,ART}^{k;CC/ECT\&UTT} + \rho^k + \mu(t)\right] {LA_{SL}}_{ai}^k\,. \tag{18}$$

Note that we use the shorthand $\rho^0 \equiv 0 \equiv \sigma^0$. The quantity $\delta_{CD4-thresh}^k(t)$ is defined according to the ART guidelines in place at that time, taking the value 1 if CD4 category k is within CD4 eligibility guidelines at the time, and 0 if not. If $\delta_{CD4-thresh}^k(t) = 1$ then individuals progress to ART (T), whereas if $\delta_{CD4-thresh}^k(t) = 0$ they progress to pre-ART (J). CD4 eligibility guidelines are given below. The force of infection terms are defined in terms of infectivity (λ), relative infectivities denoted (in general) i, partner change rates and riskiness of sexual behaviour (c_i and ψ_i respectively), assortativity θ, and proportion of sex acts with individuals not in the targetted country π, as in Cori et al. [1]. All of the above quantities, in addition to the simulation start date t_{start}, are defined in Table A (parameters varied in the analysis) and Table B (all other quantities in the model) and values given where appropriate.

Table A. Parameters varied in calibration

Parameter	Interpretation	Range	Reference
t_{start}	Date of simulation start	1978.6 - 1988.5	Based on date of first recorded AIDS case in South Africa [7]
λ	Basic infectivity, defined as the infectivity of a middle risk individual when not in acute infection, late infection or on ART	0.05 - 1.00 year^{-1}	Range was suitable to produce multiple fits; same values as in [1]
i_{ART}	Relative infectivity on ART, compared to basic infectivity	0.04 - 0.5	Lower limit from [8]. Upper limit based on lower estimate from [9] that only 50% of those on ART are virally suppressed, and assuming no effectiveness of ART if unsuppressed
f_1, f_2, f_3	% in higher (f_1), middle (f_2) and lower (f_3) risk groups. Note that $f_3 = 1 - f_1 - f_2$	High: 1-19% Middle: 1-79%	Limits chosen to allow exploration of two risk model with high and low or high and medium risk only, along with all scenarios in-between
c_1	Higher risk partner change rate	1.5 - 15.0 year^{-1}	Lower limit 1.5× greater than middle risk partner change rate (to maintain risk category distinction). High value chosen to explore extreme scenarios
θ	Sexual mixing assortativity	1-99%	Full range used to be conservative, as in [1]

Continued on next page

Table A – *Continued from previous page*

Parameter	Interpretation	Range	Reference
$\tau^{k;CC}_{ret,ART}$	Defined as $\tau^{k;CC}_{ret,ART} = \alpha^{CC}_{ret,ART}\rho^k$; rate of return to ART in CC context	$\alpha^{CC}_{ret,ART}$: 0.10 - 3.77	Range chosen so that between 5 and 80% of those with CD4 < 200 cells/μL return to ART within one year of drop-out
σ^4	Rate of HIV associated mortality from CD4 < 200 cells/μL when on ART	0.0368 - 0.0424 year^{-1}	Based on [10], assuming ART initiation at 35 years
$\phi_{SL\,ART}$	LTFU rate from second line ART	0.15 - 0.24 year^{-1}	Minimum from an ART drop-out meta-analysis [11]. Maximum from [12]

Table B. Definition of other terms in equations (1) **to** (18)

Parameter	Interpretation	Value	Reference
$\text{FOI}_{ai}(t)$	Force of infection for gender a and risk group i	Dynamically calculated	See below; method from [1]
i_p	Relative infectivity of acute stage	26.04	[13]
i_{heal}	Relative infectivity during circumcision healing period	0.385	[14, 15]
i^1, i^2, i^3, i^4	Relative infectivity of CD4 stages	1, 1, 1, 2.34	[13]
$i_{initART}$	Relative infectivity when initiating ART (compartment T)	0.5	Value is minimum possible improvement in infectivity in sensitivity analysis of i_{ART}; chosen to be conservative regarding early impact of ART as in [1]
s_{heal}	Relative susceptibility to infection during circumcision healing period	0.33	[14]
s_{circ}	Relative susceptibility of circumcised males	0.40	[16–19]
$p^k_{initCD4}$	Proportion starting in CD4 stage ≥500, 350-500, 200-350, < 200 cells/μL after end of acute infection	0.58, 0.23, 0.16, 0.03	Derived in [1] from data in [20]
ρ^p	Rate of leaving acute infection	4.14 year^{-1}	[13]
ρ^1, ρ^2, ρ^3	Rate of progression from CD4 stage k to $k+1$	0.157, 0.35, 0.282 year^{-1}	[20]
ρ^4	Rate of HIV mortality from CD4 < 200 cells/μL category	0.434 year^{-1}	[21]
$\sigma^1, \sigma^2, \sigma^3$	Rate of disease progression when on ART between stages k to $k+1$	0, 0, ρ^3	See text
τ^{lab}_{casc}	CD4 staging rate - laboratory CD4 testing	8.16 year^{-1}	Median waiting times for CD4 testing, receiving results and initiating ART averaged from [22, 23]. Rate derived from these as $\tau^{lab}_{casc} = \ln(2)/\text{median time}$

Continued on next page

Table B – *Continued from previous page*

Parameter	Interpretation	Value	Reference
τ_{casc}^{POC}	CD4 staging rate - POC CD4 testing	37.5 year^{-1}	Median waiting times for CD4 testing, receiving results and initiating ART averaged from [22, 23]. Rate derived from these as $\tau_{casc}^{lab} = \ln(2)/\text{median times}$
$\tau_{ret,diag}^{k,POC/lab;CC}$	Rate determining numbers re-entering care after LTFU during diagnostic cascade, defined according to CD4 test type and CD4 count: $\tau_{ret,diag}^{k,POC/lab;CC} = p_{CD4results}^{POC/lab}\alpha_{ret,diag}\rho^k$. Note when ART ineligible return to care rate scales with rate of leaving compartment: $p_{CD4results}^{POC/lab}\rho^k$.	$\alpha_{ret,diag} = 3.70$	Set to match ART coverage in 2012 [24]; $p_{CD4results}^{POC/lab}$ discussed below
$\tau_{ret,diag}^{k,POC/lab;ECT\&UTT}$	Rate determining numbers re-entering care after LTFU during diagnostic cascade in ECT and UTT programmes. When ART ineligible: $\tau_{ret,diag}^{k,POC/lab;ECT\&UTT} = p_{CD4results}^{POC/lab}\rho^k$	3.15 year^{-1}	Chosen so that approximately 95% of patients will return to care within a year in ECT and UTT contexts (ignoring other routes out of the compartment)
$\tau_{ret,ART}^{k;ECT\&UTT}$	Rate of return to ART from LTFU	3.15 year^{-1}	Chosen so that approximately 95% of patients will return to ART within a year in ECT and UTT contexts (ignoring other routes out of the compartment)
ψ_1, ψ_2, ψ_3	Relative rate of unprotected sexual acts within same risk group partnership, relative to partnerships between two middle risk partners; relative rate between partners of different risk groups is also set to one	2, 1, 0.5	[25]
c_2, c_3	Partner change rate, middle and lower risk categories	1, 0.1 year^{-1}	As in [1]; values chosen to retain clear distinctions between sexual risk categories, uncertainty captured by varying c_1 in a wide interval
mc_{birth}	Proportion circumcised at birth	0.76	[26, 27]
$\tau_-(t)$	Negative testing rate, determining rate of circumcision	Dynamically generated	Method from [1]
$\tau_{a,+}^{k;CC/ECT\&UTT}(t)$	Positive testing rate	Depends on context, see equations (19) and (20) and text	See text; method from [1]

Continued on next page

Parameter	Interpretation	Value	Reference
$\tau_{link}^{POC/lab}$	Rate describing how individuals go from knowing HIV status to knowing CD4 status (varies by CD4 test type)	See (21)	Details in text
τ_{suppr}	Rate of attaining viral suppression after initiating ART	8 year^{-1}	[28]
τ_{heal}	Rate of healing after circumcision procedure	26 year^{-1}	[29]
τ_{circ}	Rate of getting circumcised once decision to get circumcised has been made	26 year^{-1}	Central target value in [1]. Value not a strong predictor of outcome in [1] so not varied here.
π	Proportion of sex acts with partners outside the country	0.05	Central target value in [1]. Value not a strong predictor of outcome in [1] so not varied here.
$\phi_{diag}^{POC/lab}$	Rate of being unsuccessful in linking to care.	See (23)	Details in text
ϕ_{ART}	Rate of dropping out of ART.	0.11 year^{-1}	Central target in [1]; implicitly varied by varying rate of return to ART.
ϕ_{preART}	Rate of dropping out of pre-ART	0.84 year^{-1}	[30]
$\tau_{preART-ART}$	Rate of initiating ART from pre-ART when individual becomes eligible	1.39 year^{-1}	Based on recommendation to CD4 test once every six months in pre-ART [31]
$\tau_{SL\,ART}$	Rate of first-line ART failure and entering second-line ART	0.03 year^{-1}	[32]
$\beta(t)$	Per-adult birth rate at time t	$\beta(t) = \beta_0(t)/\kappa$	Method from [1]
$\beta_0(t)$	Per-capita birth rate at time t	Varies	UN population division
κ	Proportion of adults	0.70	UN population division
$N(t)$	Population size at time t	Varies	UN population division
$\mu(t)$	Death rate at time t	Generated to match population estimates	Method from [1]

S1.3 Further details on parameterisation and model structure

S1.3.1 Demography

Demographics are treated as in [1]. Briefly, individuals are born according to bi-weekly projections of national birth rates starting at t_{start} and extending to the simulation finish date. Those born in a particular year enter the susceptible adult population after a 15 year delay. 50% of births are of each gender (hence the factor of 0.5 in equations (1), (4) and (5)). Death rates $\mu(t)$ are constructed to reflect projections of mortality for the country as a whole [1].

S1.3.2 Force of infection and sexual risk group characteristics

The details of the force of infection (FOI$_{ai}$) calculation are as in [1], with the only modification to incorporate the additional state variables in the present analysis. Briefly, each HIV-positive individual has a basic infectivity λ, defined as the infectivity of a middle risk individual when not in acute infection, late infection or on ART. This is modified by multiplying by factors related to condom use (ψ_i), stage of HIV infection, and whether the individual

is on ART or not. No difference is assumed by gender, and only heterosexual sex is modelled. The relative infectivities for the acute and chronic HIV stage k are i_p and i^k respectively. When an individual starts ART there is an initial period (state variable T) lasting on average $1/\tau_{suppr}$, when viral load is not fully reduced and the corresponding relative infectivity is the minimum possible reduction in infectivity when on ART: $i_{initART} = 0.5$. Individuals who remain on ART after this time are assumed to have infectivity reduced by a factor i_{ART} with the upper bound for effectiveness based on the HPTN 052 study [8], and the lower bound taking into account that a large fraction of individuals on ART may not be fully virally suppressed.

Individuals mix assortatively with those in the same risk group, with the degree of assortativity determined by the parameter θ. Higher risk groups are characterised by higher partner change rates ($c_1 > c_2 > c_3$), while they also have different levels of condom use ψ_i. Assortative partnerships have an associated relative number of instantaneous, unprotected sex acts: $\psi_1 = 2$, $\psi_2 = 1$, $\psi_3 = 0.5$ which implies that higher risk individuals have twice as many unprotected sexual encounters as middle risk individuals and so on. The remainder, $1 - \theta$, of partnerships occur between any risk group (random mixing). Such partnerships have an average duration of c_2^{-1}. The relative number of instantaneous, unprotected sex acts is set equal to the middle risk group ($\psi_2 = 1$).

A separate country is simulated without any intervention. A proportion π of sexual mixing occurs with this country. As it was shown in [1] that this has minimal impact on the epidemiology it is not discussed further here.

S1.3.3 HIV testing

HIV testing begins in 2004 to coincide with increased ART availability in South Africa. HIV testing is taken to consist of two components: a standard rate of testing (in all three care contexts), and enhanced testing (in the ECT and UTT care contexts only), with the latter corresponding to the extra testing efforts that could be achieved, for example, by country-wide home-based HIV testing campaigns. In equation (10), $\tau_{a,+}^{k;CC/ECT\&UTT}$ denotes the rate of HIV testing (and receiving HIV test results) for HIV-positive individuals who collect their HIV results. This is comprised either of just a background HIV testing term ($\tau_{a,+}^{k;CC}$, for the CC context) or background HIV testing term plus additional measures in the ECT and UTT care contexts ($\tau_{a,+}^{k;ECT\&UTT}$). These are modelled as follows.

The annual number of HIV tests is determined by data from Johnson et al. [24] and implemented as a per-year testing rate: $n_{tests}(t)$. The testing numbers given there are implemented in the model by linearly interpolating between them and allowing a certain percent increase per year after mid-2011, set to 2% per year (compounded). The number of positive tests is determined by the proportion who are positive but whose status is unknown (i.e. there is no assumption of bias toward or against testing in HIV-positive or negative individuals [24]). To test the sensitivity of the model to this assumption of 2% annual increase in the number of HIV tests during the period mid-2011 to 2015, we ran the model with values ranging from -3.9% to 6.1%. However, the relative change in infections averted was only 3% of the range derived in the full infections averted analysis, showing that it was not a sensitive parameter, and it was, therefore, not varied in calibration.

We next try to quantify the relative HIV testing frequencies of individuals in different CD4 categories, on the assumption that those with lower CD4 counts are more likely to have symptoms and, therefore, be tested and diagnosed. A recent meta-analysis showed that the mean CD4 count of recently diagnosed HIV-positive individuals is 251 cells/μL in Sub-Saharan Africa [33]. Although no result specifically for South Africa was presented, we used the given result for Sub-Saharan Africa as a guide to the average CD4 count of recently tested individuals. By assigning approximate values to the CD4 count at time of testing for the four categories (tests in categories k=1,2,3,4 are assigned CD4 count values of 500, 350, 200, 50 respectively) the relative testing frequencies of the four categories in the model are fixed at ζ_k =1:2:3:5 relative to the highest CD4 count category. Without more precise data to fix the numbers testing at different CD4 counts this ensured the model partially captured the known effect that the majority of HIV tests occur at low CD4 counts. A bias in female to male HIV testing ($G_{fm} = 1.69$) was also added [24] so that undiagnosed HIV-positive women test more quickly.

Mathematically, the rate of HIV testing at a given time t is given by the annual testing rate $n_{tests}(t)$, multiplied by the proportion who are HIV-positive, the relative probability of testing by gender and the relative probability

of testing by CD4 category:

$$\tau_{f,+}^{k;CC} = n_{tests}(t)\frac{I}{I+S}\frac{G_{fm}}{1+G_{fm}}\frac{\zeta_k}{\sum_k \zeta_k I_f^k}, \tag{19}$$

$$\tau_{m,+}^{k;CC} = n_{tests}(t)\frac{I}{I+S}\frac{1}{1+G_{fm}}\frac{\zeta_k}{\sum_k \zeta_k I_m^k} \tag{20}$$

where:

$$I_a^k \equiv \sum_{i=1}^{3} I_{ai}^k \quad \text{and} \quad I \equiv \sum_{a\in\{m,f\}} \sum_{k=1}^{4} I_a^k.$$

In the ECT and UTT contexts, additional HIV testing is included, such as might be achieved by implementing intensive home-based or workplace-based HIV testing campaigns.

S1.3.4 CD4 staging

In order to capture the CD4 staging process, we model two separate components: firstly, that some individuals will be LTFU at the CD4 staging step; and secondly individuals who are not LTFU will take some time to link to care. We represent this through the three compartments H, C, and L. The flows from H to L, and from H to C, aim to capture the proportion of individuals who are LFTU, and who link to care, respectively. Individuals move out of H at a high rate, and enter either C or L according to the proportion who successfully CD4 stage or become LTFU respectively. Those who move from H to C then wait in C for a time, modelling the delay due to CD4 staging. Following this they move either to J or T, depending on eligibility.

The rate of leaving the HIV status known compartment H and entering the CD4 count known compartment C is:

$$\tau_{link}^{POC/lab} = \tau_{\text{immediate}} \times p_{CD4results}^{POC/lab} \times p_{ART-initiate}^{POC/lab}, \tag{21}$$

where $\tau_{\text{immediate}}$ is discussed below, and is such that the flow of individuals is nearly instantaneously split between those who are LTFU and those who move to C ($\tau_{\text{immediate}}$ is such that 90% move out of H every time-step). For an individual to be CD4 staged rather than LTFU, they need to return for their CD4 results and then initiate ART. The two proportions $p_{CD4results}^{POC/lab}$ and $p_{ART-initiate}^{POC/lab}$ give, respectively, the proportion who successfully return for their CD4 results once CD4 tested, and the proportion who begin ART having received their CD4 test results. The superscripts indicate whether a laboratory or POC CD4 test was done.

Meta-analysis of laboratory and POC CD4 parameters. The proportions $p_{CD4results}^{POC/lab}$ and $p_{ART-initiate}^{POC/lab}$ define the difference between the POC CD4 scenario and laboratory CD4 comparator in our study. To evaluate these, a meta-analysis of available trial data was carried out as follows. Relevant studies were found by a review of the literature and reference to a previous meta-analysis [34]. From these studies, two proportions were extracted for both POC and laboratory CD4 test types: the proportion that returned for their CD4 results having been CD4 tested; and the proportion that initiated ART after receipt of CD4 results. Not all studies reported all of the proportions. The raw data from the studies used are listed in Table C, along with brief descriptions of the studies.

To combine the results from the different studies, proportions were first transformed using Freeman-Tukey double arcsine transformations [35]. These transformed proportions were pooled using the DerSimonian-Laird random effects model to produce a most likely transformed proportion and associated 95% confidence intervals [36]. The transformed proportions were then inverse transformed using the Miller inversion formula [37]. All calculations were done in Microsoft Excel 2013. The results of this meta-analysis are presented in the results section of the main text, Table 1. The product of the proportions for either POC CD4 or laboratory CD4 test in Table 1 (main text) define the proportion of individuals that either move to C and are about to enter treatment (see equation (21)), or move to L and are considered LTFU (see equation (23) below).

Studies never reported a rate of returning for results or initiating ART but reported a proportion returning in a specified follow-up period (see Table C). Follow-up periods were, however, highly heterogenous or not reported for the quantities in which we were interested. In particular, several studies reported the time between HIV test and ART initiation only, and did not break this time down into the steps of the CD4 staging process as required

here. Consequently, we model LTFU as an instantaneous process and used the proportions directly to inform $p_{CD4results}^{POC/lab}$ and $p_{ART-initiate}^{POC/lab}$. This implicitly introduces the assumption that the study follow-up time was such that all individuals who were going to return for CD4 results did so, and those who did not can be classified as LTFU. Finally, to account for the heterogeneity of the studies combined, proportions were varied uniformly within the 95% confidence intervals obtained in the meta-analysis.

Since rates were not combined in the meta-analysis, there is no time unit to associate with the movement from H to C. Consequently, individuals were instantly placed into category C. The time-delay associated with the CD4 staging process is then applied to those successfully staged through a mean waiting period in compartment C of $1/\tau_{casc}^{POC/lab}$.

One final note about our modelling of the CD4 staging process concerns the step from HIV diagnosis to undergoing a CD4 test. No attempt was made to parameterise the loss between HIV diagnosis and going for a CD4 test since this depends on the HIV test setting (be it home-based, in a clinic or otherwise). As we only sought to model improvements due to the speed with which CD4 test results can be made available following a POC CD4 test, only the loss between CD4 test and receiving CD4 test results (and changes in ART initiation following this) were modelled. Formally this means that the numbers entering compartment H have both received an HIV-positive test result and undergone CD4 testing (but not received results yet). Due to the considerable uncertainty in total numbers of HIV tests used to calculate the rate at which individuals enter compartment H, it was considered acceptable to neglect this source of LTFU.

Approach to sensitivity analysis. The proportions $p_{CD4results}^{POC/lab}$ and $p_{ART-initiate}^{POC/lab}$ derived in the meta-analysis are varied uniformly and independently across their 95% confidence interval ranges. This is in contrast to a more standard approach using odds ratios (ORs). In a scenario where a study is a trial reporting on the proportions successfully staging in laboratory testing and POC CD4 testing arms, the two proportions could be combined to form an odds ratio (OR). Unfortunately, this approach could not be taken because not all studies considered in the meta-analysis reported on paired results between laboratory and POC CD4 testing [38–40]. In a pooled OR approach, such “one-sided” data points would have to be removed. It was not, ultimately, deemed appropriate to remove studies given the small overall number of studies available (eight).

Moreover, we aimed to explore as wide a range of improvements due to introduction of POC CD4 testing as possible, in order to acknowledge lack of studies, and between-study heterogeneity. We found that removing the aforementioned papers (such that pooled ORs could be calculated) narrowed the range of values explored. To show this, “implied” ORs can be calculated for our simulation. This is done by using the proportions from the Latin hypercube sampling and combining them in the following way to calculate the OR for each simulation:

$$OR = \frac{p^{POC}/(1-p^{POC})}{p^{lab}/(1-p^{lab})} \tag{22}$$

Taking the simulation inputs, the median and 95% confidence intervals for the ORs can be inferred:

$$OR_{CD4results} = 6.6\ (2.2 - 27.1),$$
$$OR_{ART-initiate} = 1.5\ (0.8 - 3.2).$$

Alternatively, we can calculate ORs from the literature values, after elimination of the appropriate papers, using standard meta-analysis techniques. This leads to the following ORs:

$$OR_{CD4results} = 5.3\ (3.7 - 7.6),$$
$$OR_{ART-initiate} = 1.8\ (0.8 - 3.3).$$

The original approach, with its wider range for $OR_{CD4results}$, is more conservative: it explores less effective and more effective implementations of POC CD4 testing. As the ultimate impact of better or worse implementations cannot be known in advance (for example, more effective schemes combined with high LTFU may have reduced cost-effectiveness) it is preferable to retain the wider range of values to explore such possibilities. This allows us to acknowledge the considerable heterogeneity between studies and renders our results more robust to the uncertainty in the impact of introducing POC CD4 testing in a given setting.

Table C. Papers used in meta-analysis of impact of POC CD4 testing versus laboratory CD4 testing. Note not all papers were used in all meta-analysis results.

Lead author and year	Country	Setting	Outline of study	Follow-up period*	No. get CD4 results/No. CD4 tested		No. initiate ART/No. get CD4 results (of those ART eligible)	
					POC	Lab	POC	Lab
Faal, 2011 [41]	South Africa	Urban clinic	Randomised control study of effect of POC vs. laboratory CD4 testing at a clinic in Johannesburg	3 months	124/124	50/112	28/43	9/13
Jani, 2011 [22]	Mozambique	Rural and peri-urban clinics	Retrospective cohort study of impact of on-site POC CD4 testing in four clinics across Maputo and Sofala provinces	90 days (CD4 test), 60 days (ART initiate)	345/437	214/492	94/144	57/93
Larson, 2012 [38]	South Africa	Mobile clinic	Pseudo-randomised trial of impact of POC vs. laboratory CD4 testing at a mobile clinic that operated in public places across Johannesburg	N/A	263/279	-	-	-
Larson, 2013 [42]	South Africa	Urban clinic	Retrospective study of a POC CD4 pilot vs. baseline rates of patient attrition at Themba Lethu clinic, Johannesburg	16 weeks	-	-	135/273	103/223
Losina, 2010 [40]	South Africa	Rural and urban clinics	Prospective cohort study of LTFU in standard care between HIV test and CD4 staging (KwaZulu-Natal)	N/A	-	212/248	-	-
Matambo, 2012 (poster) [43]	South Africa	Mobile clinic	Retrospective analysis of impact of POC vs. laboratory CD4 testing in a migrant worker population with access to mobile clinic (Limpopo province)	N/A	594/735	951/2171	188/226	193/380
Patten, 2013 [23]	South Africa	Youth clinics	Before/after observational study examining impact of POC CD4 testing on retention in care of youths (Khayelitsha)	N/A	275/304	183/272	18/36	19/44
van Rooyen, 2013 [39]	South Africa	Home based testing	Analysis of impact of POC CD4 testing on patient retention in existing home-based care and testing (HBCT) programme in KwaZulu-Natal	6 months	-	-	19/35	-

*N/A (not available): information on follow-up time not given in study for outcomes recorded here

S1.3.5 Treatment

Once an individual has entered the pre-treatment compartment C, they can subsequently either initiate ART, if eligible according to guidelines at the time, or else enter pre-ART. ART eligibility guidelines used here were as follows: before 2011.5, CD4<200 cells/μL initiate ART [3]; 2011.5 to 2015.0, CD4 <350 cells/μL , [4]. At the start of 2015, eligibility criteria change to either CD4<500 in CC and ECT context [5], or immediate ART regardless of CD4 count in UTT context. Guidelines changes (whereby individuals in compartment k become eligible for ART) are implemented progressively by increasing the appropriate HIV testing rate (τ_+^k) from 0%, at the start of the guideline change, to 100% of the possible number treated, six months later.

Treatment effectiveness in preventing transmission. Individuals on ART are assumed to have reduced infectivity. We use a range of values for this parameter to account for the fact that not all patients on ART may be fully virally suppressed [44]. For the upper limit of ART effectiveness we use the results from Cohen et al. [8]. The lower limit is based on recent UNAIDS estimates that as little as half of those receiving ART in Sub-Saharan Africa, may be virally suppressed [9], and we assume that those not virally suppressed have no reduced infectivity. This produces a very conservative lower estimate for ART effectivity of 50%.

Disease progression on ART. The impact of ART, aside from reduced infectivity, is to increase life expectancy. Johnson et al. [10] have estimated life expectancy of adults on ART by age at ART initiation. Since these estimates were computed at a time when ART eligibility was restricted to those with low CD4 counts, we use these estimates to inform the average time individuals spend in the final CD4 compartment when on treatment. We use the life expectancy estimates for individuals starting ART at 35 years of age, averaged over men and women, as the mean time spent in the final CD4 compartment when on ART ($1/\sigma^4$).

Since analogous life expectancies from higher starting CD4 counts are not known (ART eligibility only got expanded in 2011 [4]) the additional benefits of starting at CD4 count 200 < CD4 $\leq$ 350 cells/μL are less well characterised. We assume similar benefits to starting in the final CD4 category and for simplicity allow disease progression at the untreated rate until the individual reaches the final CD4 compartment: $\sigma^3 = \rho^3$. In other words all the benefits of ART occur in the final CD4 count category. To simulate the benefits of very early ART initiation, we assumed $\sigma^1 = \sigma^2 = 0$ year^{-1}. Work on the original model suggests that projections over short timescales are insensitive to large changes in rates of progression on treatment [1]. Therefore, the values of σ^1, σ^2 and σ^3 were not varied.

Second line ART. 3% of the patients on first-line ART are assumed to experience treatment failure each year, and enter the more expensive second line therapy [32, 45]. The rate of retention in second line care is varied between 14% and 21% [12, 46]. The rates of return from second line drop out are varied simultaneously with first line treatment.

S1.4 Loss to follow-up

Loss to follow-up at CD4 staging or pre-ART In S1.3.4, we discuss how individuals in H (infected, known status) move to either LTFU (L) or pre-treatment (C). Those who are unsuccessful in linking to care enter L at a rate

$$\phi_{diag}^{POC/lab} = \tau_{\text{immediate}} \times \left(1 - p_{CD4results}^{POC/lab} \times p_{ART-initiate}^{POC/lab}\right). \tag{23}$$

Those on pre-ART can also become LTFU, entering the same compartment L at a rate ϕ_{preART}. They must re-enter care through a new CD4 test via compartment C. ϕ_{preART} is such that losses to follow-up in pre-ART are 57% per year following [30, 47].

Return to care when LTFU prior to initiating ART. Individuals who drop out at CD4 staging or during pre-ART are treated in the same way. The rate at which these individuals link back into care depends on context

and for ART eligible individuals is given by:

$$\tau_{ret,diag}^{k,POC/lab;CC} = p_{CD4results}^{POC/lab} \alpha_{ret,diag} \rho^k, \tag{24}$$

$$\tau_{ret,diag}^{k,POC/lab;ECT\&UTT} = p_{CD4results}^{POC/lab} \rho^k. \tag{25}$$

$\alpha_{ret,diag}\rho^k$ represents the rate at which individuals return to care after a period of LTFU (assumed to vary with time spent in a particular CD4 compartment). In order to capture the additional delays due to the need to receive another CD4 test when returning to care, we scale this rate by $p_{CD4results}^{POC/lab}$ (the proportion of individuals who return for CD4 results following either POC or laboratory CD4 test), leading to equations (24) and (25). Note the proportion $p_{CD4results}^{POC/lab}$ is varied uniformly within the 95% confidence intervals obtained in the meta-analysis. ART ineligible individuals are assumed to link back to care slower (at a rate proportional to the time spent in their particular CD4 count compartment $p_{CD4results}^{POC/lab}\rho^k$).

The quantity $\alpha_{ret,diag}$ is chosen such that the total number on ART rises to over 2 million by 2012 [48, 49]. The value was first set during analysis of a particular set of parameters. In subsequent analyses, instead of varying this value, a requirement was built into calibration that the number on ART be over 2 million by mid-2012 to prevent running simulations with an unrealistically low number of treated patients (and thus unrealistically low projections of future treatment costs).

LTFU from ART. Individuals on ART who drop out of treatment enter compartment LA (LA_{SL} when on second-line treatment) at a rate $\phi_{ART} = 0.11$ year^{-1} [1]. Once LTFU they undergo HIV disease progression and mortality at the same rate as prior to initiating ART. The rate of return from LA and LA_{SL} are assumed to be the same: $\tau_{ret,ART}^{k;CC/ECT\&UTT}$, however, this rate of return differs according to the context (either CC or ECT/UTT).

In the CC context, which models the current care situation in South Africa, there is a lack of data to inform this quantity $\tau_{ret,ART}^{k;CC}$ [50]. The only assumption made in the model is that the rate of return to ART varies proportionately to the rate of disease progression: $\tau_{ret,ART}^{k;CC} = \alpha_{ret,ART}^{CC}\rho^k$. This allows for a decrease in care seeking behaviour at the highest CD4 counts (when the disease is assumed to be pre-clinical) [51]. The constant of proportionality $\alpha_{ret,ART}^{CC}$ is varied in calibration; it is chosen such that between 5% and 85% return (approximately) within a year of being LTFU from the lowest CD4 count compartment.

In the ECT and UTT care contexts the rates of return for all ART-eligible groups are calculated so that approximately 95% return within a year: $\tau_{ret,ART}^{k;ECT\&UTT} = 3.15$ year^{-1}. The rates of return in both these contexts are equal across all CD4 categories because individuals are assumed to return due to enhanced follow-up efforts, and not because of health-related problems.

S1.5 Calibrating the model

2 million iterations of the model were run by sampling parameters from a Latin hypercube [52] with uniform prior ranges specified in Table A. Each parameter set then had to pass two calibration steps, described below, to be deemed an 'acceptable' parameter set: it had to be consistent with UNAIDS HIV prevalence estimates, and the number of individuals on ART had to be consistent with data.

To calibrate the model, epidemic trajectories were simulated for each of these parameter sets up to 2013, and the resulting model HIV prevalence was compared to the latest annual UNAIDS estimates of prevalence over the period 1993 to 2013 [53]. If the model HIV prevalence was within twice the confidence intervals defined by the UNAIDS estimate for all years, the parameter set was taken to be calibrated to the UNAIDS estimates. A second requirement for each model simulation was that at least 2 million individuals were on ART by mid-2012 [49], in order to ensure that costs were calculated with adequate numbers of individuals on treatment. This further reduced the number of acceptable parameter combinations.

To evaluate which parameters were responsible for the success or failure of a parameter set in the calibration process, a logistical model was used to model the probability of success or failure of a parameter set to satisfy the demands of the calibration. Due to the small number of successful parameter sets, a bias reduced logistical model was used, using the **R** package 'brglm' [54]. Those parameters that were not significantly associated with the success or failure of a parameter set were varied along with the cost and CD4 testing parameter ranges in

cost-effectiveness analysis, see Table E and main text Table 1 respectively. As these were the parameters that, by definition, did not contribute to the successful calibration of the simulation, they were not expected to have a major impact on the ICERs and infections averted. However, with no support for choosing a particular value for these parameter sets they were allowed to vary representing the uncertainty in those parameters.

S1.6 Cost-effectiveness analysis

S1.6.1 Approach to calculating DALYs

DALYs are the sum of the years lived with disability (YLD) and years of life lost (YLL) due to a particular disease. DALYs averted compares the DALYs (in a given treatment and care context) of a simulation run with POC CD4 testing to a simulation run with laboratory testing. YLDs are calculated by multiplying the average annual compartmental occupancies in a given year by the standardised DALY weights (see Table D for DALY weights used). This gives a measure of YLD as per current Global Burden of Disease guidelines [55,56]. YLLs are the sum of all years of life lost by all individuals who would otherwise be alive had POC CD4 testing been implemented.

Table D. DALY weights used in the economic analysis. All values taken from Eaton et al. [57].

Disease stage	DALY weight (per year)
Not infected	0
HIV: CD4 $<$ 200 cells/μL	0.547
HIV: 200 $\leq$ CD4 $<$ 350 cells/μL	0.221
HIV: CD4 $\geq$ 350 cells/μL	0.053
HIV: on ART	0.053

S1.6.2 Approach to calculating costs

The cost approach was adopted from Eaton et al., [57]. In this, posterior means were calculated for the various costs in South Africa using a Bayesian meta-analysis approach. These are reported in Table E (see table for details of inflation adjustment). Initiation costs are dealt with as follows. As the key part of the analysis, CD4 tests costs are calculated according to modality (POC or laboratory) and are added after CD4 testing. Since both laboratory and POC CD4 test arms have the same numbers of HIV tests, these do not need an associated cost as it would drop out of the calculation.

Re-initiation does not incur a cost. While not strictly accurate, from a modelling perspective the uncertainty in the rate with which individuals leave and re-enter ART is so great that adding a cost to the movement between LTFU and ART could potentially have a significant impact on cost. The inaccuracy introduced by ignoring this cost can be considered offset by supposing the total ART cost to include an amortised cost of re-initiation for each patient (averaged over all patients). We implicitly, therefore, make allowance for the fact that re-initiation costs (along with ARV delivery costs) are uncertain by including a "variation in ART costs" parameter in sensitivity analysis, see Table E.

S1.6.3 Comments on how this approach fits into the GBD framework

Calculations of DALYs as performed by the Global Burden of Disease (GBD) group [55] have changed in recent years. Here we consider how our approach to calculating DALYs fits into their approach and in particular how the structure of the model led to a particular way of calculating YLLs.

YLD calculations have changed recently from being a measure of disease incidence to a measure of current disease prevalence [55]. Following the GBD approach, we calculate YLDs by multiplying together prevalence, disability weight and time-step size and summing over all compartments for a given year.

The 2010 approach to calculating YLL (Global Burden of Disease - GBD - group [55]) is to sum the number of deaths in a year and multiply by the years of life lost compared to a reference life expectancy of approximately 86.4 years (depending on age), based on the maximum global life expectancy (Japan). In our analysis, however, we are interested only in the short term gains, not the burden of disease [60]. Furthermore, the lack of age stratification

Table E. Costs used in the economic analysis. Values from Eaton et al., [57], multiplied by 84.8%, according to 1st June 2012 to 1st June 2015 inflation of South African Rand (ZAR) inflation (accounting for currency fluctuations between ZAR and USD) .

**Cost**	**Value (2015 $US)**	**Reference**
First line ARV cost	120	[58]
Second line ARV cost	310	[58]
ARV provision cost	358	[57]
Pre-ART (CD4 $\geq$ 350 cells/μL)	174	[57]
Pre-ART (200 $\leq$ CD4 $<$ 350 cells/μL)	202	[57]
Pre-ART (CD4 $<$ 200 cells/μL)	304	[57]
Untreated health care costs (CD4 $\geq$ 350 cells/μL)	11	[57]
Untreated health care costs (200 $\leq$ CD4 $<$ 350 cells/μL)	39	[57]
Untreated health care costs (CD4 $<$ 200 cells/μL)	142	[57]
End-of-life-care	136	[57]
Lab CD4 test	6.24	[59]
POC CD4 test	15.88 - 32.32	[59]
Variation in ART costs	$\pm$ 20%	NA
Variation in non-ART costs	$\pm$ 20%	NA
Discount rate	0.1 - 7.0%	NA

makes it difficult to assign a number for YLL to premature HIV deaths in the model. We, therefore, simply add 1 YLL to the DALYs in the year in which they occur. Specifically, in the year a death occurs, a value of between 0 and 1 is recorded for the proportion of the year lost due to premature mortality (0 for a death at the end of December, 1 for a death at the beginning of January, etc.). This is the YLL contribution to the DALY that year due to a premature death. In all subsequent years, the death is recorded as 1 YLL. An individual alive due to the introduction of POC CD4 testing (but not otherwise) thus contributes one DALY averted each year they remain alive (less the YLD contribution of that individual in the POC CD4 simulation). This corresponds to accounting for DALY contributions in the year in which they occur, in other words a pure prevalence approach to the calculation of YLL, rather than all YLL being considered at the point of death [56].

S1.6.4 Probabilistic cost-effectiveness analysis: further methods

For each of the 71 acceptable sets of parameters derived from the calibration, a further 250 parameter sets were produced by exploring ranges of values for the CD4 and cost parameters (see methods, main text). Infections averted and ICERs comparing POC CD4 and laboratory CD4 testing for 1 and 3 year projections were calculated for each of the 71 $\times$ 250 parameter sets, and for each care context.

Significant predictors of the infections averted and ICERs associated with introducing POC CD4 testing in place of laboratory testing were assessed by linear regression: all varied parameters were used as predictors in the multivariable linear regression. In place of using $p^{POC/lab}_{CD4results}$ and $p^{POC/lab}_{ART-initiate}$ directly, we defined the differences between the proportions to capture the improvement due to the introduction of POC CD4 testing:

$$\Delta p_{CD4results} = p^{POC}_{CD4results} - p^{lab}_{CD4results},$$
$$\Delta p_{ART-initiate} = p^{POC}_{ART-initiate} - p^{lab}_{ART-initiate},$$

and used these in the linear regressions in place of the proportions themselves. The proportion of variance due to each parameter (or derived parameter) was then computed using the Lindeman, Merenda and Gold (lmg) method [61], as implemented in the **R** package ‘relaimpo’ [62]. Standardised coefficients are reported. These are equal to Pearson’s correlation coefficients and thus give a measure of the one-way sensitivity of the results to changes in that parameter [63]. Provided the model R^2 is large, such that non-linear interaction terms are not required to explain the variance, then the standardised regression coefficients can be directly used to infer which parameters drive the variation in outcomes.

References

1. Cori A, Ayles H, Beyers N, Schaap A, Floyd S, Sabapathy K, et al. HPTN 071 (PopART): a cluster-randomized trial of the population impact of an HIV combination prevention intervention including universal testing and treatment: mathematical model. PLoS One. 2014;9(1):e84511.

2. Boulle A, Bock P, Osler M, Cohen K, Channing L, Hilderbrand K, et al. Antiretroviral therapy and early mortality in South Africa. Bulletin of the World Health Organization. 2008;86(9):678–687.

3. WHO. Scaling up antiretroviral therapy in resource-limited settings; 2002. Available from: http://www.who.int/hiv/pub/prev_care/en/ScalingUp_E.pdf [accessed 28 November 2015].

4. WHO. Antiretroviral therapy for HIV infection in adults and adolescents: recommendations for a public health approach; 2010. Available from: http://apps.who.int/iris/bitstream/10665/44379/1/9789241599764_eng.pdf [accessed 28 November 2015].

5. WHO. Consolidated guidelines on the use of antiretroviral drugs for treating and preventing HIV infection: recommendations for a public health approach; 2013. Available from: http://apps.who.int/iris/bitstream/10665/85321/1/9789241505727_eng.pdf?ua=1 [accessed 28 November 2015].

6. WHO. Guideline on when to start antiretroviral therapy and on pre-exposure prophylaxis for HIV; 2015. Available from: http://apps.who.int/iris/bitstream/10665/186275/1/9789241509565_eng.pdf [accessed 12 April 2016].

7. Ras G, Simson I, Anderson R, Prozesky O, Hamersma T. Acquired immunodeficiency syndrome. A report of 2 South African cases. S Afr Med J. 1983;64:140–142.

8. Cohen MS, Chen YQ, McCauley M, Gamble T, Hosseinipour MC, Kumarasamy N, et al. Prevention of HIV-1 infection with early antiretroviral therapy. N Engl J Med. 2011;365(6):493–505.

9. UNAIDS. Access to antiretroviral therapy in Africa: status report on progress towards the 2015 targets; 2013. Available from: http://www.unaids.org/sites/default/files/media_asset/20131219_AccessARTAfricaStatusReportProgresstowards2015Targets_en_0.pdf [accessed 1 February 2015].

10. Johnson LF, Mossong J, Dorrington RE, Schomaker M, Hoffmann CJ, Keiser O, et al. Life expectancies of South African adults starting antiretroviral treatment: collaborative analysis of cohort studies. PLoS Med. 2013;10(4):e1001418.

11. Rosen S, Fox MP, Gill CJ. Patient retention in antiretroviral therapy programs in sub-Saharan Africa: a systematic review. PLoS Med. 2007;4(10):e298.

12. Fox MP, Maskew M, MacPhail AP, Long L, Brennan AT, Westreich D, et al. Cohort profile: the Themba Lethu clinical cohort, Johannesburg, South Africa. Int J Epidemiol. 2012;42(2):430–439.

13. Hollingsworth TD, Anderson RM, Fraser C. HIV-1 transmission, by stage of infection. J Infect Dis. 2008;198(5):687–693.

14. Mehta SD, Gray RH, Auvert B, Moses S, Kigozi G, Taljaard D, et al. Does sex in the early period after circumcision increase HIV-seroconversion risk? Pooled analysis of adult male circumcision clinical trials. AIDS. 2009;23(12):1557–1564.

15. Wawer MJ, Makumbi F, Kigozi G, Serwadda D, Watya S, Nalugoda F, et al. Circumcision in HIV-infected men and its effect on HIV transmission to female partners in Rakai, Uganda: a randomised controlled trial. Lancet. 2009;374(9685):229–237.

16. Auvert B, Taljaard D, Lagarde E, Sobngwi-Tambekou J, Sitta R, Puren A, et al. Randomized, controlled intervention trial of male circumcision for reduction of HIV infection risk: the ANRS 1265 Trial. PLoS Med. 2005;2(11):e298.

17. Bailey RC, Moses S, Parker CB, Agot K, Maclean I, Krieger JN, et al. Male circumcision for HIV prevention in young men in Kisumu, Kenya: a randomised controlled trial. Lancet. 2007;369(9562):643–656.

18. Gray RH, Kigozi G, Serwadda D, Makumbi F, Watya S, Nalugoda F, et al. Male circumcision for HIV prevention in men in Rakai, Uganda: a randomised trial. Lancet. 2007;369(9562):657–666.

19. Mills E, Cooper C, Anema A, Guyatt G. Male circumcision for the prevention of heterosexually acquired HIV infection: a meta-analysis of randomized trials involving 11,050 men. HIV Med. 2008;9(6):332–335.

20. Lodi S, Phillips A, Touloumi G, Geskus R, Meyer L, Thiébaut R, et al. Time from human immunodeficiency virus seroconversion to reaching CD4+ cell count thresholds < 200, < 350, and < 500 cells/mm^3: assessment of need following changes in treatment guidelines. Clin Infect Dis. 2011;53(8):817–825.

21. Badri M, Bekker LG, Orrell C, Pitt J, Cilliers F, Wood R. Initiating highly active antiretroviral therapy in sub-Saharan Africa: an assessment of the revised World Health Organization scaling-up guidelines. AIDS. 2004;18(8):1159–1168.

22. Jani IV, Sitoe NE, Alfai ER, Chongo PL, Quevedo JI, Rocha BM, et al. Effect of point-of-care CD4 cell count tests on retention of patients and rates of antiretroviral therapy initiation in primary health clinics: an observational cohort study. Lancet. 2011;378(9802):1572–1579.

23. Patten GE, Wilkinson L, Conradie K, Isaakidis P, Harries AD, Edginton ME, et al. Impact on ART initiation of point-of-care CD4 testing at HIV diagnosis among HIV-positive youth in Khayelitsha, South Africa. J Int AIDS Soc. 2013;16:18518.

24. Johnson L, Dorrington R, Rehle T, Jooste S, Bekker LG, Wallace M, et al. THEMBISA version 1.0: A model for evaluating the impact of HIV/AIDS in South Africa. Cape Town, Centre for Infectious Disease Epidemiology and Research; 2014. Available from: http://webdav.uct.ac.za/depts/epi/publications/documents/THEMBISA%20version%201.0.pdf [accessed 2 February 2015].

25. Powers KA, Ghani AC, Miller WC, Hoffman IF, Pettifor AE, Kamanga G, et al. The role of acute and early HIV infection in the spread of HIV and implications for transmission prevention strategies in Lilongwe, Malawi: a modelling study. Lancet. 2011;378(9787):256–268.

26. Sismanidis C, Moulton LH, Ayles H, Fielding K, Schaap A, Beyers N, et al. Restricted randomization of ZAMSTAR: a 2× 2 factorial cluster randomized trial. Clin Trials. 2008;5(4):316–327.

27. Ayles HM, Sismanidis C, Beyers N, Hayes RJ, Godfrey-Faussett P. ZAMSTAR, The Zambia South Africa TB and HIV Reduction Study: design of a 2 x 2 factorial community randomized trial. Trials. 2008;9:63.

28. Matthews GV, Sabin CA, Mandalia S, Lampe F, Phillips AN, Nelson MR, et al. Virological suppression at 6 months is related to choice of initial regimen in antiretroviral-naive patients: a cohort study. AIDS. 2002;16(1):53–61.

29. Kigozi G, Gray RH, Wawer MJ, Serwadda D, Makumbi F, Watya S, et al. The safety of adult male circumcision in HIV-infected and uninfected men in Rakai, Uganda. PLoS Med. 2008;5(6):e116.

30. Du Toit E, Van Schalkwyk C, Dunbar R, Jennings K, Yang B, Coetzee D, et al. Missed opportunities for retention in pre-ART care in Cape Town, South Africa. PLoS One. 2014;9(5):e96867.

31. WHO. Clinical guidelines for the management of HIV and AIDS in adults and adolescents; 2010. Available from: http://www.who.int/hiv/pub/guidelines/south_africa_art.pdf [accessed 3 March 2015].

32. WHO. Prioritizing second-line antiretroviral drugs for adults and adolescents: a public health approach; 2007. Available from: http://www.who.int/hiv/pub/meetingreports/Second_Line_Antiretroviral.pdf [accessed 3 March 2015].

33. Siedner MJ, Ng CK, Bassett IV, Katz IT, Bangsberg DR, Tsai AC. Trends in CD4 count at presentation to care and treatment initiation in Sub-Saharan Africa, 2002-2013: a meta-analysis. Clin Infect Dis. 2014;60(7):1120–7.

34. Wynberg E, Cooke G, Shroufi A, Reid SD, Ford N. Impact of point-of-care CD4 testing on linkage to HIV care: a systematic review. J Int AIDS Soc. 2014;17(1):18809.

35. Freeman MF, Tukey JW. Transformations related to the angular and the square root. Ann Math Stat. 1950;21(4):607–611.

36. DerSimonian R, Laird N. Meta-analysis in clinical trials. Control Clin Trials. 1986;7(3):177–188.

37. Miller JJ. The inverse of the Freeman–Tukey double arcsine transformation. Am Stat. 1978;(32):138–138.

38. Larson B, Schnippel K, Ndibongo B, Xulu T, Brennan A, Long L, et al. Rapid point-of-care CD4 testing at mobile HIV testing sites to increase linkage to care: an evaluation of a pilot program in South Africa. J Acquir Immune Defic Syndr. 2012;61(2):e13–e17.

39. van Rooyen H, Barnabas RV, Baeten JM, Phakathi Z, Joseph P, Krows M, et al. High HIV testing uptake and linkage to care in a novel program of home_based HIV counseling and testing with facilitated referral in KwaZulu-Natal, South Africa. J Acquir Immune Defic Syndr. 2013;64(1):e1–e8.

40. Losina E, Bassett IV, Giddy J, Chetty S, Regan S, Walensky RP, et al. The "ART" of linkage: pre-treatment loss to care after HIV diagnosis at two PEPFAR sites in Durban, South Africa. PLoS One. 2010;5(3):e9538.

41. Faal M, Naidoo N, Glencross DK, Venter WD, Osih R. Providing immediate CD4 count results at HIV testing improves ART initiation. J Acquir Immune Defic Syndr. 2011;58(3):e54–e59.

42. Larson BA, Schnippel K, Brennan A, Long L, Xulu T, Maotoe T, et al. Same-day CD4 testing to improve uptake of HIV care and treatment in South Africa: point-of-care is not enough. AIDS Res Treat. 2013;2013:Article ID 941493.

43. Matambo T, Hildebrand K, Mwongera C, Wilkinson L, Van Cutsem G, Bauernfeind A, et al. Impact of a mobile HIV/TB service on pre-ART retention among migrant farm-workers in Musina, South Africa. Poster session presented at: XIX International Aids Conference; 22-27 July 2012; Washington, DC. Available from: http://aids2012.msf.org/wp-content/uploads/2012/06/Impact-of-a-Mobile-HIV-TB-Service-on-Pre-ART-Poster.pdf.

44. Fox MP, Shearer K, Maskew M, Meyer-Rath G, Clouse K, Sanne I. Attrition through multiple stages of pre-treatment and ART HIV care in South Africa. PLoS One. 2014;9(10):e110252.

45. Granich R, Kahn JG, Bennett R, Holmes CB, Garg N, Serenata C, et al. Expanding ART for treatment and prevention of HIV in South Africa: estimated cost and cost-effectiveness 2011-2050. PLoS One. 2012;7(2):e30216.

46. Fox MP, Ive P, Long L, Maskew M, Sanne I. High rates of survival, immune reconstitution, and virologic suppression on second-line antiretroviral therapy in South Africa. J Acquir Immune Defic Syndr. 2010;53(4):500–506.

47. Lessells RJ, Mutevedzi PC, Cooke GS, Newell ML. Retention in HIV care for individuals not yet eligible for antiretroviral therapy: rural KwaZulu-Natal, South Africa. J Acquir Immune Defic Syndr. 2011;56(3):e79–e86.

48. WHO in partnership with UNICEF and UNAIDS. Global update on HIV treatment 2013: results, impact and opportunities; 2013. Available from: http://www.unaids.org/sites/default/files/media_asset/20130630_treatment_report_en_0.pdf [accessed 28 November 2015].

49. Simbayi L, Shisana O, Rehle T, Onoya D, Jooste S, Zungu N, et al.. South African national HIV prevalence, incidence and behaviour survey, 2012; 2014. Available from: http://www.hsrc.ac.za/uploads/pageContent/4565/SABSSM%20IV%20LEO%20final.pdf [accessed 1 March 2015].

50. Klein DJ, Bershteyn A, Eckhoff PA. Dropout and re-enrollment: implications for epidemiological projections of treatment programs. AIDS. 2014;28:S47–S59.

51. Hoffman S, Wu Y, Lahuerta M, Kulkarni SG, Nuwagaba-Biribonwoha H, El Sadr W, et al. Advanced disease at enrollment in HIV care in four sub-Saharan African countries: change from 2006 to 2011 and multilevel predictors in 2011. AIDS. 2014;28(16):2429–2438.

52. McKay MD, Beckman RJ, Conover WJ. Comparison of three methods for selecting values of input variables in the analysis of output from a computer code. Technometrics. 1979;21(2):239–245.

53. UNAIDS. Prevalence data for South Africa; 2014. Available from: http://www.unaids.org/en/regionscountries/countries/southafrica [accessed 24 December 2014].

54. Kosmidis I. brglm: Bias reduction in binary-response GLMs; 2007. R package version 0.5-6.

55. Murray CJ, Ezzati M, Flaxman AD, Lim S, Lozano R, Michaud C, et al. GBD 2010: design, definitions, and metrics. Lancet. 2013;380(9859):2063–2066.

56. Schroeder SA. Incidence, prevalence, and hybrid approaches to calculating disability-adjusted life years. Popul Health Metr. 2012;10:19.

57. Eaton JW, Menzies NA, Stover J, Cambiano V, Chindelevitch L, Cori A, et al. Health benefits, costs, and cost-effectiveness of earlier eligibility for adult antiretroviral therapy and expanded treatment coverage: a combined analysis of 12 mathematical models. Lancet Glob Health. 2014;2(1):e23–e34.

58. Médecins Sans Frontières. Untangling the web of antiretroviral price reductions - 17th edition; 2014. Available from: http://www.msfaccess.org/sites/default/files/MSF_UTW_17th_Edition_4_b.pdf [accessed 3 March 2015].

59. Cassim N, Coetzee LM, Schnippel K, Glencross DK. Estimating implementation and operational costs of an integrated tiered CD4 service including laboratory and point of care testing in a remote health district in South Africa. PLoS One. 2014;9(12):e115420.

60. Rushby JF, Hanson K. Calculating and presenting disability adjusted life years (DALYs) in cost-effectiveness analysis. Health Policy Plann. 2001;16(3):326–331.

61. Lindeman RH, Merenda PF, Gold RZ. Introduction to bivariate and multivariate analysis. Harlow, UK: Longman Higher Education; 1980.

62. Grömping U. Relative importance for linear regression in R: the package relaimpo. J Stat Softw. 2006;17(1):1–27.

63. Helton JC, Davis F. Illustration of sampling-based methods for uncertainty and sensitivity analysis. Risk Analysis. 2002;22(3):591–622.
