# Supplementary material for: Impact and Cost-Effectiveness of Point-Of-Care CD4 Testing on the HIV Epidemic in South Africa
Source: PLoS One. 2016 Jul 8;11(7):e0158303. doi: 10.1371/journal.pone.0158303 (PMC4938542; doi:10.1371/journal.pone.0158303)

S2 Supporting Information. Additional results

S2.1 Calibrating the model

Details of the calibration process are given in S1.5 of S1 Supporting Information. Of the 2 million parameter sets simulated, we obtained 71 acceptable fits according to our calibration procedure, whereby prevalence was constrained to lie within twice the UNAIDS prevalence estimates confidence intervals and 2 million were required to be on ART by mid-2012. HIV prevalence and incidence curves for all simulations are given in Fig C.

Fig C. Prevalence and incidence of calibrated parameter sets. Dots indicate the UNAIDS estimates confidence intervals (inner circles) and twice the confidence intervals (outer crosses) on the left panel.

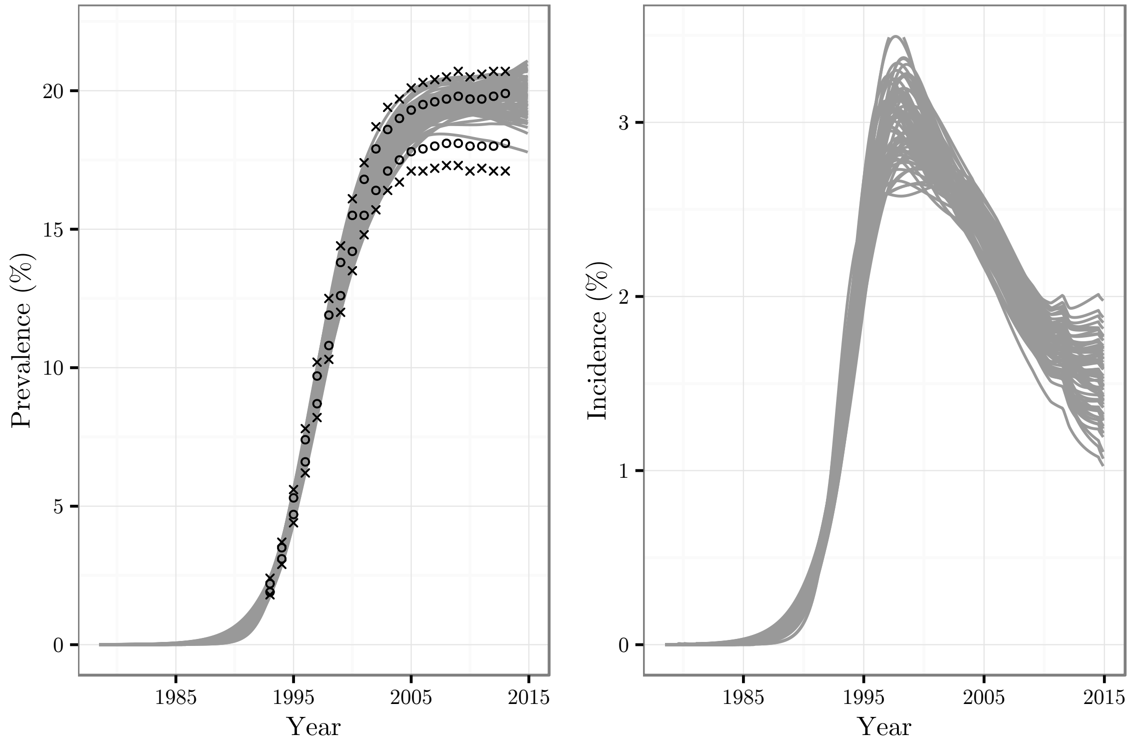

The logistic regression showed that the key parameters in simulating the epidemic were: epidemic start date, proportion in higher and middle risk groups, higher risk group partner change rate, basic infectivity, assortativity and second line ART drop out ($p < 0.001$ in all cases except second line ART dropout for which $p = 0.048$). Due to the borderline significance for the second line drop-out rate, this parameter was varied in the cost-effectiveness analysis, along with all the remaining parameters (see S1 Supporting Information, Table A) not shown to be instrumental in determining whether the epidemic curve plausibly represented South Africa.

S2.1.1 Results after calibrating more closely to UNAIDS estimates

The prescription of fitting within twice the UNAIDS estimates was used because the confidence intervals are extremely narrow before 1998 and fitting within only 1× confidence intervals over the whole period lead to only 4 parameter sets satisfying the calibration criteria. Even if this criterion is relaxed such that fits are only required to be within the 1× confidence intervals after 1998 (and are required to be within 2× confidence intervals before this) still only 15 parameter sets were accepted, see Fig D.

To test whether only including those fits that did pass closer to the UNAIDS mean prevalence values would affect the outcomes, we removed all fits that passed outside of the 1x confidence intervals after 1998. This corresponded to including 4618 (26%) of the simulations originally performed. Doing so we recalculated the probability of the

Fig D. Prevalence and incidence of calibrated parameter sets. Only shown are the best-fitting simulations, defined as those that remain within the UNAIDS prevalence estimates' confidence intervals after 1998 (shown as circles on the left panel); simulations may be within twice the confidence intervals up to 1998.

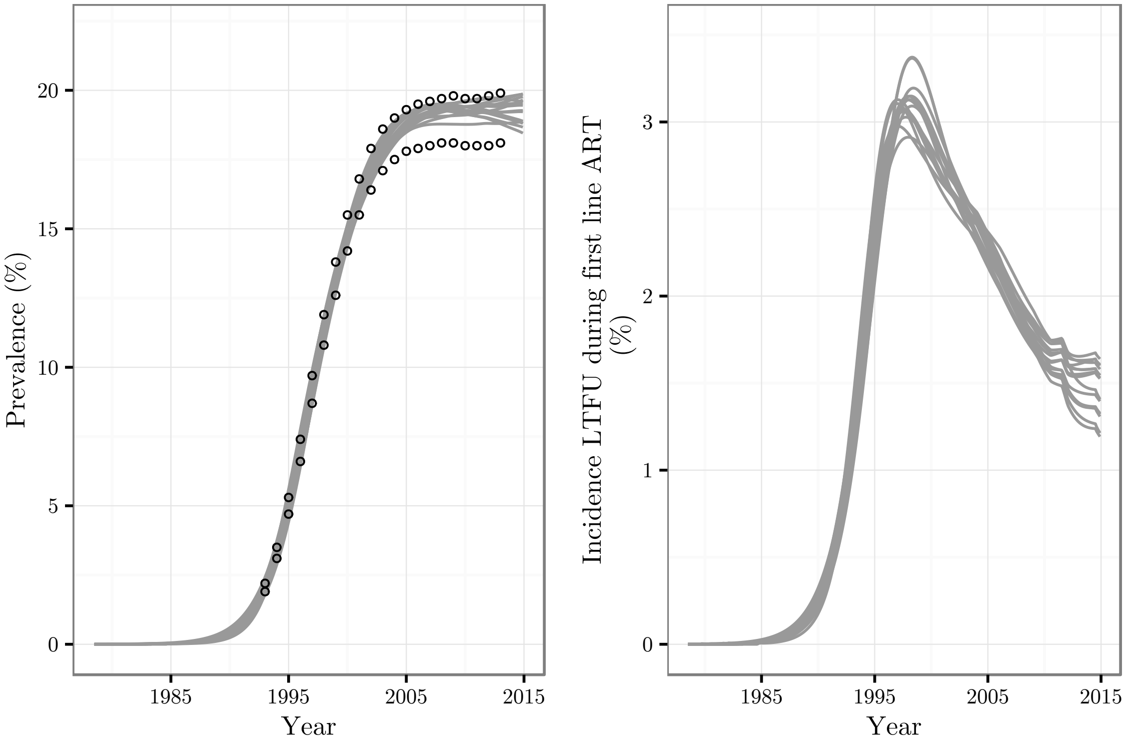

intervention being cost-effective. After 1 year 99.8% of simulations are cost-effective in CC context, 35.5% in ECT and 1.7% in UTT. After 3 years these values rise to 100%, 95.3% and 86.6%. Cost-effectiveness acceptability curves for this subset of the results are shown in Fig E. These values are extremely similar to the original values.

S2.2 Sensitivity analysis

The standardised regression coefficients and proportion of variance explained by the varied parameters for the 1 and 3 year projections are shown in Table F and Table G respectively. Using non-standardised regression coefficients (to keep parameters in their original units) linear relationships between infections averted (IA) and ICER are listed (from the linear regressions); only those variables that contribute greater than 10% to the R^2 are included in the relationship. In general this leads to including treatment effectiveness and probability of getting CD4 results in the linear approximation to the IA and probability of getting CD4 results, cost of ART (including drug cost and delivery cost with logistic markup) and (in some cases) cost of CD4 test in the linear approximation to the ICER.

1 year:

$$\mathrm{IA}_{\mathrm{CC}} \simeq -3.83 + 8.38\Delta p_{\mathrm{CD4results}} + 4.07\eta_{treat}$$
$$\mathrm{IA}_{\mathrm{ECT}} \simeq -9.54 + 16.13\Delta p_{\mathrm{CD4results}} + 10.11\eta_{treat}$$
$$\mathrm{IA}_{\mathrm{UTT}} \simeq -13.71 + 17.28\Delta p_{\mathrm{CD4results}} + 16.22\eta_{treat}$$
$$\mathrm{ICER}_{\mathrm{CC}} \simeq 4267.34 - 3462.62\Delta p_{\mathrm{CD4results}} - 251.47c_{\mathrm{ART\ cost}} + 61.09c_{\mathrm{POC\text{-}test}}$$
$$\mathrm{ICER}_{\mathrm{ECT}} \simeq 6261.17 - 8620.32\Delta p_{\mathrm{CD4results}} + 238.37c_{\mathrm{ART\ cost}} + 145.96c_{\mathrm{POC\text{-}test}}$$
$$\mathrm{ICER}_{\mathrm{UTT}} \simeq 9223.99 - 10536.97\Delta p_{\mathrm{CD4results}} - 288.41c_{\mathrm{ART\ cost}} + 151.85c_{\mathrm{POC\text{-}test}}$$

Fig E. Cost-effectiveness acceptability curves. These curves show the probability that introduction of POC CD4 testing compared to laboratory CD4 testing is cost-effective at a range of decision rule thresholds for the 1 year projection (left) and 3 year projection (right) for the subset of fits that fall within the 1x UNAIDS confidence intervals after 1998. The colours correspond to (left to right within each plot): grey - current care (CC) context, orange - enhanced counselling and testing (ECT), blue - universal test and treat (UTT). The dashed line shows South African GDP per capita.

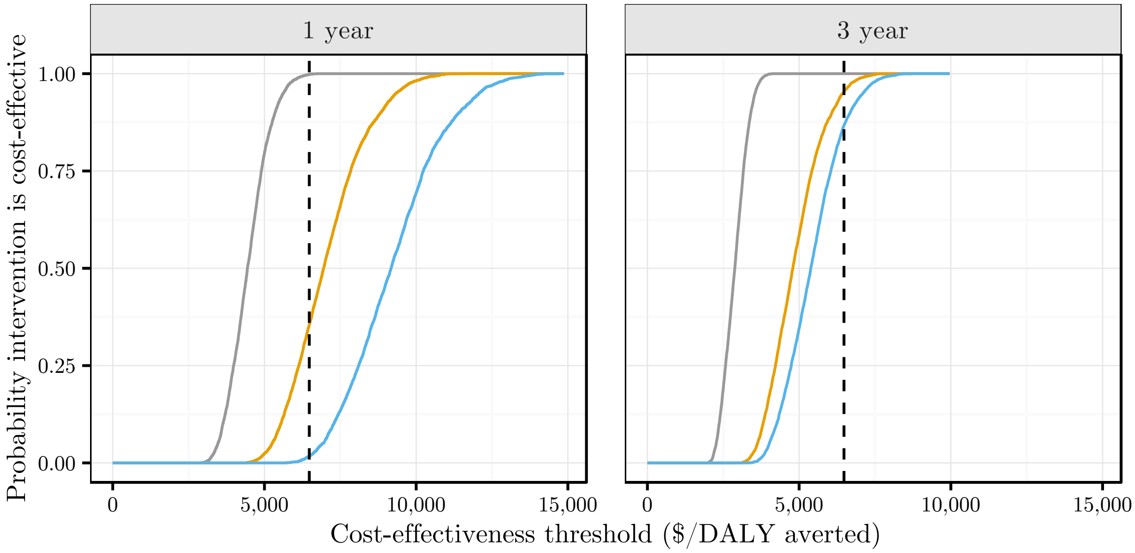

3 year:

$$\mathrm{IA}_{\mathrm{CC}} \simeq -6.42 + 9.94\Delta p_{\mathrm{CD4results}} + 7.09\eta_{treat}$$
$$\mathrm{IA}_{\mathrm{ECT}} \simeq -9.11 + 12.53\Delta p_{\mathrm{CD4results}} + 9.68\eta_{treat}$$
$$\mathrm{IA}_{\mathrm{UTT}} \simeq -12.65 + 11.42\Delta p_{\mathrm{CD4results}} + 15.14\eta_{treat}$$
$$\mathrm{ICER}_{\mathrm{CC}} \simeq 3021.66 - 1730.29\Delta p_{\mathrm{CD4results}} - 169.8c_{\mathrm{ART\ cost}}$$
$$\mathrm{ICER}_{\mathrm{ECT}} \simeq 5126.12 - 6488.85\Delta p_{\mathrm{CD4results}} + 490.06c_{\mathrm{ART\ cost}} + 67.97c_{\mathrm{POC\text{-}test}}$$
$$\mathrm{ICER}_{\mathrm{UTT}} \simeq 6183.6 - 6196.89\Delta p_{\mathrm{CD4results}} - 180.96c_{\mathrm{ART\ cost}}$$

Table F. Results of linear regression in the cost-effectiveness analysis for the 1 year projection

Parameter	Range		Outcome of linear regression (by context)											
	Most optimistic	Most pessimistic	CC				ECT				UTT			
			Inf. Av.		ICER		Inf. Av.		ICER		Inf. Av.		ICER	
			Sl.	Var.	Sl.	Var.	Sl.	Var.	Sl.	Var.	Sl.	Var.	Sl.	Var.
Median return to ART time	3.77	0.1	-0.03	0.2	0.00	0.0	0.02	0.0	0.00	0.0	0.01	0.0	0.00	0.0
Treatment effectiveness	0.04	0.50	0.52	26.5	-0.02	0.0	0.60	35.2	-0.05	0.3	0.72	52.2	-0.06	0.4
Percentage second line drop out	0.15	0.24	0.00	0.0	0.00	0.0	0.00	0.0	0.00	0.0	0.00	0.0	0.00	0.0
Years of life treated HIV	0.0368	0.0424	0.01	0.0	0.00	0.0	0.01	0.0	0.00	0.0	0.01	0.0	0.00	0.0
Improvement in proportion receiving CD4 results	55.0	9.3	0.80	64.4	-0.52	27.2	0.72	51.3	-0.68	45.8	0.58	33.6	-0.66	43.5
Improvement in number starting ART	30.8	-10.4	0.05	0.3	-0.16	2.5	0.01	0.0	-0.08	0.6	0.01	0.0	-0.08	0.6
Cost of POC CD4 test	15.88	32.32	0.00	0.0	0.44	18.8	0.00	0.0	0.55	29.4	0.00	0.0	0.45	20.0
ARV supply chain multiplier	-20%	20%	0.00	0.0	0.67	45.4	0.00	0.0	0.38	15.2	0.00	0.0	0.52	27.8
Healthcare cost multiplier	-20%	20%	0.00	0.0	-0.07	0.5	0.00	0.0	0.03	0.1	0.00	0.0	-0.03	0.1
Discount rate	0.1	7.0	0.00	0.0	0.00	0.0	0.00	0.0	0.00	0.0	0.00	0.0	0.00	0.0
Proportion of variance (%)			-	91.3	-	90.4	-	86.7	-	89.2	-	86.1	-	90.9

Sl. is the standardised regression coefficient (slope) for the particular parameter; Var. is the % variation due to that parameter (adjusted R^2).

Table G. Results of linear regression in the cost-effectiveness analysis for the 3 year projection

Parameter	Range		Outcome of linear regression (by context)											
	Most optimistic	Most pessimistic	CC				ECT				UTT			
			Inf. Av.		ICER		Inf. Av.		ICER		Inf. Av.		ICER	
			Sl.	Var.	Sl.	Var.	Sl.	Var.	Sl.	Var.	Sl.	Var.	Sl.	Var.
Median return to ART time	3.77	0.1	-0.06	0.5	0.02	0.1	0.02	0.0	0.00	0.0	0.02	0.0	0.00	0.0
Treatment effectiveness	0.04	0.50	0.64	40.5	-0.07	0.5	0.64	40.2	-0.11	1.2	0.75	56.2	-0.20	3.9
Percentage second line drop out	0.15	0.24	0.00	0.0	0.00	0.0	0.00	0.0	0.00	0.0	0.00	0.0	0.00	0.0
Years of life treated HIV	0.0368	0.0424	0.01	0.0	0.00	0.0	0.01	0.0	0.01	0.0	0.01	0.0	0.01	0.0
Improvement in proportion receiving CD4 results	55.0	9.3	0.68	45.5	-0.43	18.8	0.62	38.3	-0.74	54.9	0.43	18.1	-0.66	43.0
Improvement in number starting ART	30.8	-10.4	0.04	0.2	-0.10	1.0	0.01	0.0	-0.06	0.4	0.01	0.0	-0.05	0.3
Cost of POC CD4 test	15.88	32.32	0.00	0.0	0.25	6.2	0.00	0.0	0.37	13.1	0.00	0.0	0.29	8.0
ARV supply chain multiplier	-20%	20%	0.00	0.0	0.82	67.9	0.00	0.0	0.40	16.7	0.00	0.0	0.60	36.2
Healthcare cost multiplier	-20%	20%	0.00	0.0	-0.08	0.6	0.00	0.0	0.09	0.8	0.00	0.0	-0.04	0.2
Discount rate	0.1	7.0	0.00	0.0	0.03	0.1	0.00	0.0	0.02	0.1	0.00	0.0	0.04	0.2
Proportion of variance (%)			-	86.9	-	93.6	-	78.9	-	87.4	-	74.7	-	91.1

Sl. is the standardised regression coefficient (slope) for the particular parameter; Var. is the % variation due to that parameter (adjusted R^2).

S2.3 Additional tables and graphs

Table H. Additional ICERs and infections averted

Programme	Year	Cumulative infections averted (%)			ICER ($/DALY averted)		
		**2.5%**	**Median**	**97.5%**	**2.5%**	**Median**	**97.5%**
CC	1	0.36	1.72	4.31	3356	4468	5863
	3	0.11	1.67	5.72	2199	2893	3680
ECT	1	0.17	2.65	8.78	5051	6986	9883
	3	-0.13	1.47	7.66	3503	4829	6799
UTT	1	0.11	3.09	11.47	6665	9215	12729
	3	-0.30	1.26	9.92	3896	5424	7471

Table I. Cost differences and DALYs averted

Programme	Year	DALYs averted (millions)			Total cost difference ($ billions)		
		**2.5%**	**Median**	**97.5%**	**2.5%**	**Median**	**97.5%**
CC	1	0.01	0.02	0.04	0.07	0.11	0.17
	3	0.08	0.17	0.31	0.25	0.49	0.87
ECT	1	0.02	0.03	0.05	0.14	0.22	0.34
	3	0.05	0.11	0.22	0.30	0.53	0.91
UTT	1	0.02	0.03	0.05	0.19	0.29	0.45
	3	0.05	0.11	0.22	0.32	0.58	1.06

Table J. Cost differences broken down by cost type

Programme	Year	POC CD4 test costs ($ billions)			ART costs ($ billions)		
		**2.5%**	**Median**	**97.5%**	**2.5%**	**Median**	**97.5%**
CC	1	0.01	0.03	0.04	0.05	0.09	0.15
	3	0.03	0.06	0.09	0.22	0.45	0.84
ECT	1	0.05	0.08	0.12	0.08	0.13	0.22
	3	0.07	0.12	0.19	0.15	0.31	0.62
UTT	1	0.05	0.08	0.13	0.13	0.23	0.37
	3	0.05	0.10	0.16	0.24	0.48	0.96

Fig F. Cost-effectiveness plane graph. Cost-effectiveness plane graph. Upper row is the 1 year projection, lower row is the 3 year projection. The black line corresponds to the 2015 South African GDP. The colours correspond to: grey - CC, orange - ECT, blue - UTT. Note the scales are different on the two rows.

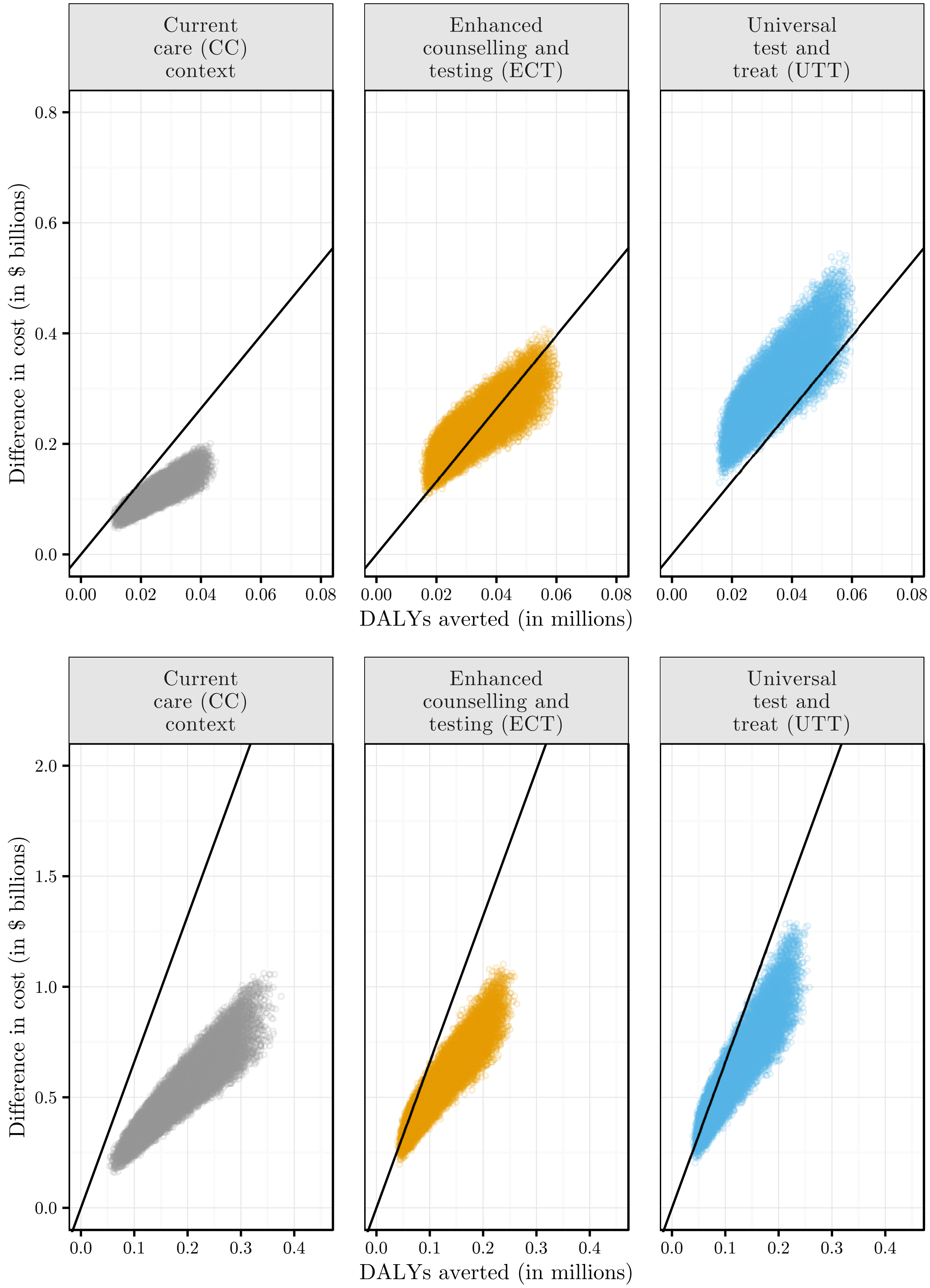

Supplement: S2 Supporting Information — contains additional results tables and graphs, including results of the linear analysis used to assess the key predictors of cost-effectiveness of introducing POC CD4 testing. (PDF) [file pone.0158303.s002.pdf]
